# Supplementary material for: An in vivo avian model of human melanoma to perform rapid and robust preclinical studies
Source: EMBO Mol Med. 2023 Jan 24;15(3):e16629. doi: 10.15252/emmm.202216629 (PMC9994476; doi:10.15252/emmm.202216629)
Supplement: Supplementary file 1 — Expanded View Figures PDF [file EMMM-15-e16629-s001.pdf]

Expanded View Figures

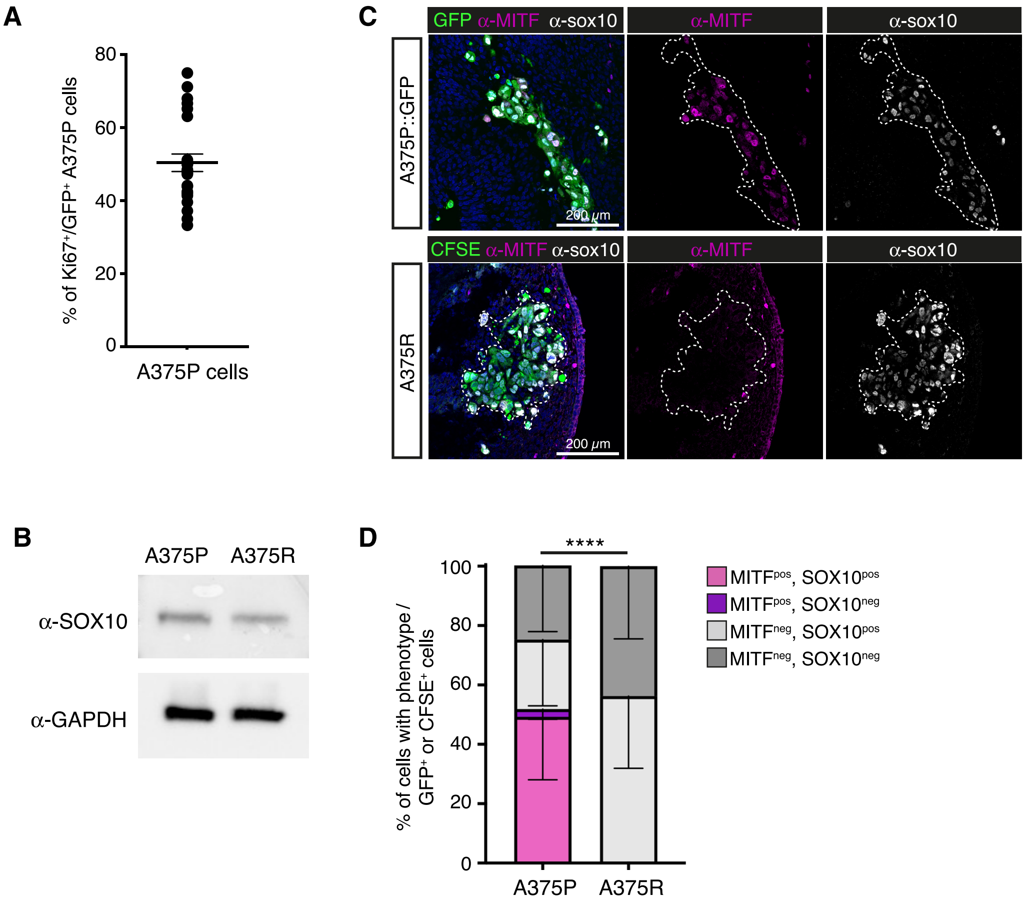

Figure EV1. Characterization of A375P and A375R cell lines in the avian graft model.

- A Quantification of Ki67 immunofluorescent staining in GFP$^+$-A375P cells, 48 h after their graft in avian embryos (related to Fig 1C) (n = 24 sections from N = 3 embryos). Bars and error bars indicate mean ± SEM.
- B Detection of SOX10 expression by Western blot in A375P and A375R cells, using GAPDH as a loading control.
- C Immunofluorescent labelling of MITF and SOX10 in HH25 avian embryos engrafted with A375P::GFP cells or A375R cells labelled with CFSE prior to the graft.
- D Quantification of the fraction of A375P (GFP$^+$, n = 23 sections from N = 3 embryos) and A375R (CFSE$^+$, n = 21 sections from N = 3 embryos) cells showing a positive or negative staining for SOX10 and/or MITF. Error bars indicate SEM. ****$P < 0.0001$, using Chi-square test comparing proportions of phenotypes in A375P versus A375R cell lines.

© 2023 The Authors

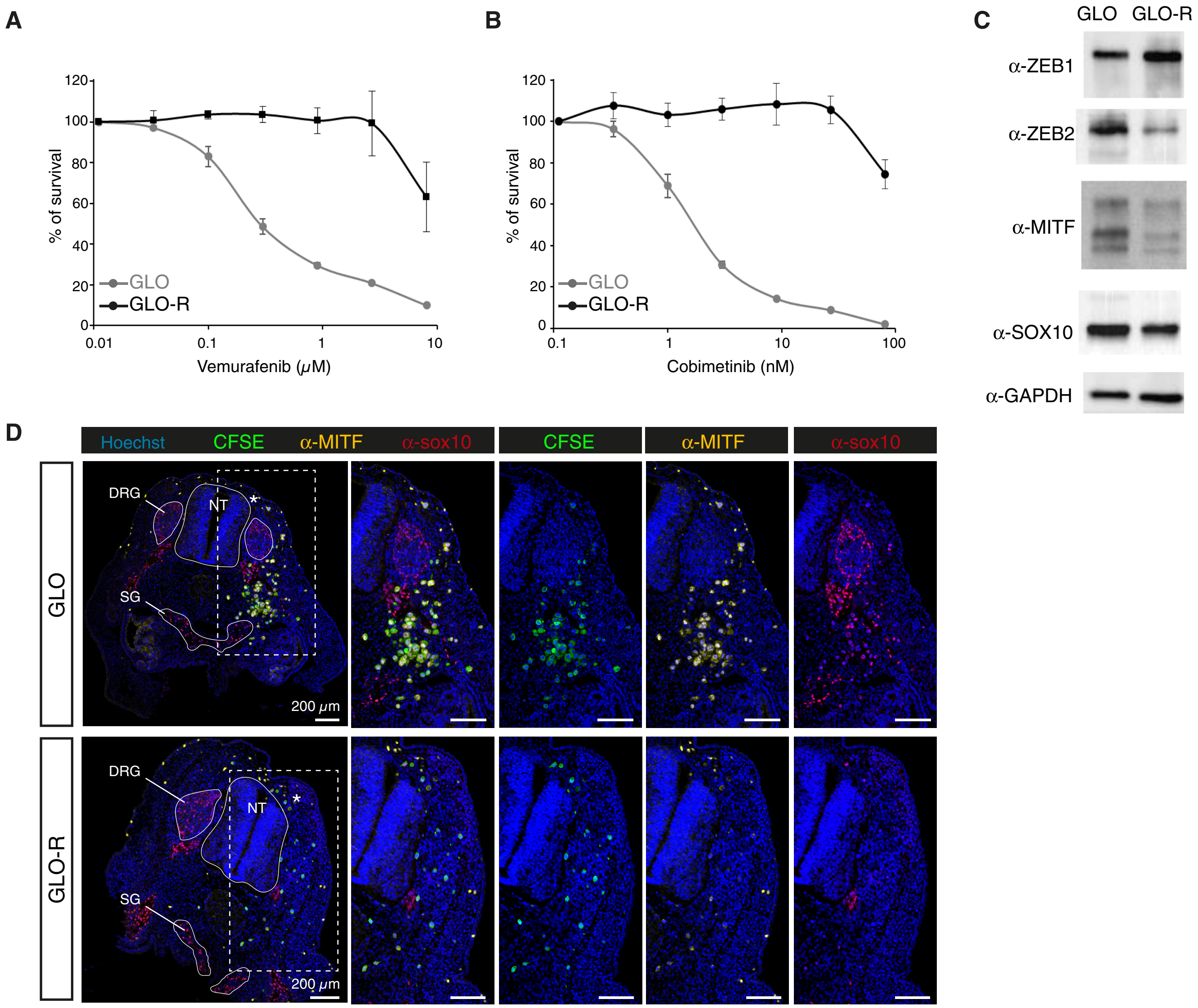

Figure EV2. Characterization of GLO and GLO-R cell lines *in vitro* and in the AVI-PDX™ model.

A, B Survival rate of GLO and GLO-R cells upon exposure to increasing doses of Vemurafenib (A) or Cobimetinib (B) for 72 h. (n = 3 technical replicates for each cell line; two biological replicates of the full experiment have been performed). Error bars show SEM.

C Detection of ZEB1, ZEB2, MITF and SOX10 expression by Western blot in GLO and GLO-R cells, using GAPDH as a loading control.

D Immunofluorescent labelling of MITF (yellow) and SOX10 (red) in HH25 avian embryos engrafted with GLO or GLO-R cells, labelled with CFSE prior to the graft. Right panels are enlargements of the left panel for GLO and GLO-R grafts.

Data information: NT: Neural Tube; DRG: Dorsal Root Ganglia; SG: Sympathetic Ganglia.

 © 2023 The Authors

Figure EV3. Effect of Vemurafenib and Cobimetinib on melanoma cells engrafted in avian embryos.

A–C Survival rate (left axis) and mean body surface area (BSA, right axis) of avian embryos injected with increasing doses of Vemurafenib (A), Cobimetinib (B) or Vorinostat (C). Each dose was administered to a minimum of 10 embryos, using excipient (NaCl) as a control. The maximum tolerated dose (MTD) was defined as the higher dose of drug associated with a survival rate higher than 75% and a mean BSA similar (i.e., non-statistically different) from embryos treated with NaCl. MTDs are indicated in red on the abscissa axis.

D Schematic diagram of the grafting procedure followed by therapy administration and assessment of therapy effect on implanted melanoma cells/biopsies.

E, F 3D views (D) and quantification of tumor volumes (G) of HH25 chick embryos engrafted with A375P or A375R cells and treated with a combination of Vemurafenib and Cobimetinib or with excipient. Scale bar: 300 µm. The numbers of embryos analyzed are indicated on the graphs.

G, H Immunostaining (G) and quantification (H) of Vemurafenib/Cobimetinib co-administration effect on avian grafts of A375P and A375R cells. Dividing cells are labeled with anti-phospho Histone 3 antibody (a-pH3) and are highlighted with red arrows. The numbers of sections analyzed are indicated on the graphs are were obtained from at least three embryos per condition.

I, J Immunostaining (I) and quantification (J) of Vemurafenib/Cobimetinib co-administration effect on avian grafts GLO and GLO-R cells. Green arrows point at dying cells with dense cytoplasmic bodies. Dividing cells are labelled with anti-phospho Histone 3 antibody (α-pH3) and are highlighted with red arrows. Scale bar: 100 µm. The numbers of sections analyzed are indicated on the graphs are were obtained from at least three embryos per condition.

Data information: Error bars indicate SEM. ***P* < 0.01, *****P* < 0.0001, ns, non-significant using Student's *t* test pr Mann-Whintey test comparing excipient versus Vemurafenib/Cobimetinib. Exact *P*-values are indicated on the graphs.

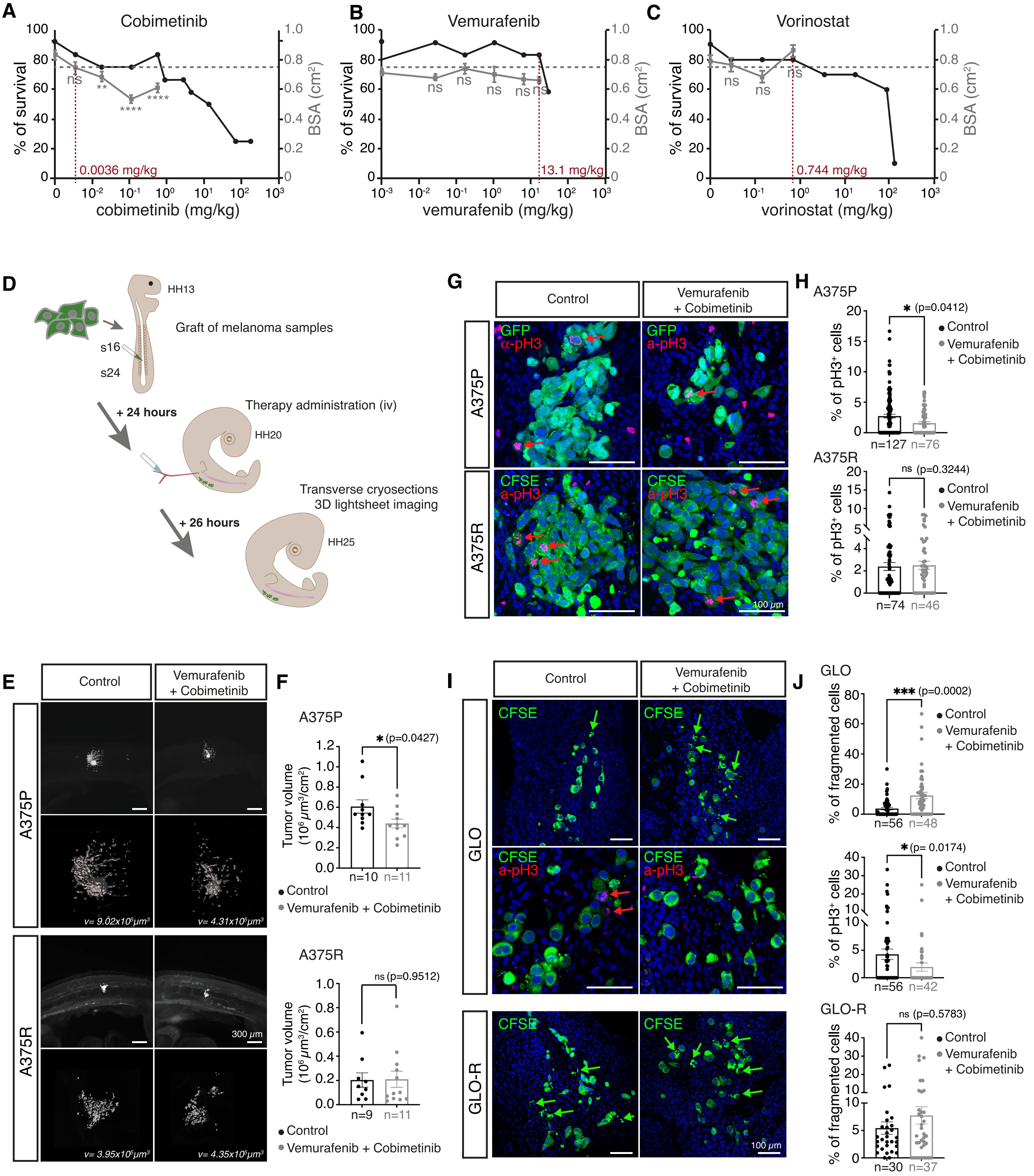

Figure EV3.

© 2023 The Authors
