# Supplementary material for: An in vivo avian model of human melanoma to perform rapid and robust preclinical studies
Source: EMBO Mol Med. 2023 Jan 24;15(3):e16629. doi: 10.15252/emmm.202216629 (PMC9994476; doi:10.15252/emmm.202216629)
Supplement: Supplementary file 2 — Table EV1 [file EMMM-15-e16629-s002.pdf]

Table EV1

Patient #	Sample characteristics						nb of grafted embryos	% tumor intake
	sex	nature	localization	stage	thickness	nb mitosis / mm^2		
Mel#1	M	primitive	scalp	I	0.40 mm	<1	11	18%
Mel#2	M	primitive	foot arch	IV	3.50 mm	4	17	100%
Mel#3	M	primitive	shoulder	II	2.05 mm	2	20	50%
Mel#4	M	primitive	back	I	0.50 mm	<1	12	83%
Mel#5	M	primitive	back	IV	5.00 mm	13	23	83%
Mel#20	M	metastasis	metastasis	IV	ND	ND	76	100%
Mel#25	M	primitive	back	IIC	6.00 mm	15	21	95%
Mel#26	F	metastasis	behind ear	IIIB	ND	ND	55	100%
Mel#27	F	primitive	calf	II	1.40 mm	<1	24	100%
Mel#28	M	metastasis	knee	IV	ND	ND	100	100%
Mel#33	M	metastasis	leg	IV	ND	ND	20	83%
Mel#34	F	metastasis	arm	IV	ND	ND	28	88%
Mel#35	M	metastasis	back	IV	ND	ND	25	100%

Table EV1: Characteristics of melanoma patient samples engrafted in avian embryos. For each patient sample, histopathological characteristics, the number of grafted avian embryos with the sample, and the tumor take rate are indicated. ND: Not Determined; M: Male, F: Female.
