# Supplementary material for: An in vivo avian model of human melanoma to perform rapid and robust preclinical studies
Source: EMBO Mol Med. 2023 Jan 24;15(3):e16629. doi: 10.15252/emmm.202216629 (PMC9994476; doi:10.15252/emmm.202216629)
Supplement: Supplementary file 3 — PDF+ [file EMMM-15-e16629-s003.pdf]

SOURCE DATA TRANSPARENT PROCESS OPEN ACCESS

EMBO Molecular Medicine

Report

An *in vivo* avian model of human melanoma to perform rapid and robust preclinical studies

Loraine Jarrosson[1,†], Stéphane Dalle[2,3,†], Clélia Costechareyre[1], Yaqi Tang[2], Maxime Grimont[2], Maud Plaschka[2], Marjorie Lacourrège[1], Romain Teinturier[1], Myrtille Le Bouar[3], Delphine Maucort-Boulch[3], Anaïs Eberhardt[2,3], Valérie Castellani[4,*,‡], Julie Caramel[2,**,‡] & Céline Delloye-Bourgeois[4,***,‡]

Abstract

Metastatic melanoma patients carrying a BRAF^{V600} mutation can be treated with a combination of BRAF and MEK inhibitors (BRAFi/MEKi), but innate and acquired resistance invariably occurs. Predicting patient response to targeted therapies is crucial to guide clinical decision. We describe here the development of a highly efficient patient-derived xenograft model adapted to patient melanoma biopsies, using the avian embryo as a host (AVI-PDX™). In this *in vivo* paradigm, we depict a fast and reproducible tumor engraftment of patient samples within the embryonic skin, preserving key molecular and phenotypic features. We show that sensitivity and resistance to BRAFi/MEKi can be reliably modeled in these AVI-PDX™, as well as synergies with other drugs. We further provide proof-of-concept that the AVI-PDX™ models the diversity of responses of melanoma patients to BRAFi/MEKi, within days, hence positioning it as a valuable tool for the design of personalized medicine assays and for the evaluation of novel combination strategies.

Keywords avian model; melanoma; patient-Derived Xenografts; preclinical oncology; targeted therapies
Subject Categories Cancer; Development; Skin
DOI 10.15252/emmm.202216629 | Received 22 July 2022 | Revised 20 December 2022 | Accepted 23 December 2022 | Published online 24 January 2023
EMBO Mol Med (2023) 15: e16629

Introduction

Cutaneous malignant melanoma is an aggressive form of skin cancer arising from melanocytes. Despite recent advances in targeted therapies and immunotherapies for the treatment of metastatic melanoma, nearly 60% of patients still develop resistance, necessitating the development of new therapeutic strategies (Dummer *et al*, 2020; Herrscher & Robert, 2020). Metastatic melanoma (MM) patients carrying a BRAF^{V600} mutation (50% of cases) can be treated with a combination of BRAF and MEK inhibitors (BRAFi/MEKi), but innate (40%) or acquired resistance invariably occurs (Larkin *et al*, 2014).

Increasing evidence suggests that resistance to BRAFi/MEKi is not only mediated by genomic alterations, but also involves phenotypic adaptations through transcriptional and epigenetic processes (Hugo *et al*, 2016; Rambow *et al*, 2018; Marine *et al*, 2020). Cellular plasticity achieved through epithelial-to-mesenchymal (EMT)-like processes contributes to intra-tumor heterogeneity (ITH) in melanoma and fosters the ability of cancer cells to adapt to treatment (Rambow *et al*, 2019; Tang *et al*, 2020). Such reversible phenotypic transitions between a proliferative/differentiated and invasive/stem-like state (Hoek *et al*, 2008), are reminiscent of the features acquired upon delamination of the embryonic neural crest from which melanocytes originate (Mort *et al*, 2015). Indeed, melanoblasts originate from neural crest cells (NCCs) that transit through a SOX10-positive melanoblast/glial bipotent progenitor state. Specified melanoblasts acquire MITF (microphthalmia-associated transcription factor) expression, mostly migrating dorsolaterally from the dorsal neural tube to reach the skin, where they differentiate into melanocytes that produce the melanin pigment. MITF is a major regulator of melanoma phenotype switching (Goding & Arnheiter, 2019). Its loss induces a reprogramming towards an invasive and stem-like phenotype accounting for targeted therapy resilience. The EMT-inducing transcription factor ZEB1 also promotes the transition towards a stem-like and invasive MITF^{low} state, resistant to BRAFi/MEKi (Caramel *et al*, 2013; Richard *et al*, 2016). While biomarkers of response to targeted therapies have been proposed, robust tools able to predict patient response in a time frame compatible with the clinical decision constitute unmet medical needs.

Various *in vivo* melanoma models (mouse, fish) have been developed over the years (Patton *et al*, 2021), each presenting pros and

1 OncoFactory SAS, Faculté de Médecine et de Pharmacie, Lyon, France
2 Université de Lyon, Université Claude Bernard Lyon 1, INSERM 1052, CNRS 5286, Centre Léon Bérard, Cancer Research Center of Lyon, Lyon, France
3 Centre Hospitalier Lyon Sud, Hospices Civils de Lyon, Pierre Bénite, France
4 University of Lyon, University of Lyon 1 Claude Bernard Lyon 1, MeLiS, CNRS UMR5284, INSERM U1314, NeuroMyoGene Institute, Lyon, France
*Corresponding author. Tel: +33 426688247; E-mail: valerie.castellani@lyon.unicancer.fr
**Corresponding author. Tel: +33 426556740; E-mail: julie.caramel@lyon.unicancer.fr
***Corresponding author. Tel: +33 426688248; E-mail: celine.delloye@univ-lyon1.fr
†These authors contributed equally to this work as co-first authors
‡These authors contributed equally to this work as co-last authors

© 2023 The Authors. Published under the terms of the CC BY 4.0 license.

cons with respect to their capacity to reproduce the human disease, the tumor heterogeneity, the tumor microenvironment (TME), and to provide relevant results for chemical screens. Starting from fresh human samples, patient-derived xenograft models (PDXs) in the mouse, are hampered by their high-cost and long timeframe for evaluating treatment efficacy, limiting their application in personalized medicine programs. Organoids may prove useful for high-throughput chemical screens, but they lack crucial components of the TME and have so far not been extensively validated for melanoma (Ronteix *et al*, 2022). Testing drug efficacy on *ex vivo* tumor fragments was recently shown to hold a robust predictive capacity (Voabil *et al*, 2021). However, it requires a significant tumor size, incompatible with large drug screenings and statistical evaluation of drug anti-tumor efficacy. The development of a PDX model, requiring low amounts of tumor samples and displaying a short-timeframe of development, would thus be a valuable preclinical tool for assessing treatment efficacy in melanoma. We recently described the development of an animal model combining these key advantages for neuroblastoma and triple negative breast cancer (TNBC), using the avian embryo as a recipient organism for patient samples (Delloye-Bourgeois *et al*, 2017; Jarrosson *et al*, 2021).

Herein, starting from human melanoma cell lines and patient samples, we provide the proof-of-concept for the setting of a highly efficient and reproducible melanoma PDX model using the avian embryo as a host. We show that this miniaturized paradigm which reproduces melanoma cell phenotypes in their microenvironment, allows to assess drug combinations, and to mimic MM patient clinical responses to targeted therapies. As such, the avian embryo PDX model (AVI-PDX^{TM}) is ideally suited for testing patient tumor responses in a timeframe compatible with therapeutic decision-making by clinicians.

Results

Micrografting human melanoma cell lines in the neural crest migration path of the avian embryo

With the aim of designing an orthotopic melanoma model, we sought to target the original lineage of melanocytes, the NCCs. Indeed, during development, a subset of NCCs reach the so-called migration staging area (MSA), in which they become melanoblasts, before engaging into migration (Mort *et al*, 2015). We hypothesized that placing melanoma cells within the MSA, could offer a supportive microenvironment to foster their migration and establishment in the skin. Thus, we engrafted human melanoma cells (BRAF^{V600E}-mutated A375P cell line) stably expressing GFP (A375P::GFP) at the MSA level, in HH13 (according to Hamburger and Hamilton staging method) chick embryos, in a trunk region lying between somite 16 and somite 24 (Fig 1A). Fifty hours after engraftment, embryos were collected at the HH25 stage and the position of grafted cells was analyzed. 3D lightsheet analysis revealed that, in all embryos, A375P::GFP cells had left the graft site to settle under the skin and in deeper tissues (Fig 1B). Previous work reported that when engrafted at earlier stages of chick development, melanoma cells spontaneously differentiate and lose their tumorigenic properties (Kulesa *et al*, 2006). In contrast, a strong Ki67 immunofluorescent labeling was observed in A375P::GFP cells within the tumor mass highlighting maintenance of high proliferative activity (Figs 1C and EV1A). Grafted cells were localized within typical NCCs dorsolateral and ventrolateral migration streams towards the developing skin or in a region under the epidermis (Fig 1B and C). We then wondered whether the SOX10-MITF axis was maintained in this particular embryonic microenvironment. A375P cells are known to display a NC stem cell-like expression pattern (MITF^{low}, SOX10^{+}) as recently analyzed at the single cell level (Wouters *et al*, 2020). Consistently, immunofluorescence analyses of A375P cells in the avian embryo revealed a heterogeneous expression of MITF with typically low or negative cells, and a few positive cells, while SOX10 expression was homogeneous (Fig 1D). Thus, implantation of human melanoma cells in a selected embryonic stage and territory drives maintenance of their tumorigenic potential and tumor growth in relevant tissues.

Generation of PDX melanoma models using the avian embryo as a host (AVI-PDX^{TM})

To extend the preclinical applicability of our model, we examined the behavior of melanoma cells isolated from patient samples. Thirteen melanoma biopsies from primary or metastatic melanoma (Table EV1), were enzymatically dissociated and labelled with CarboxyFluorescein Succinimidyl Ester (CFSE) to trace their behavior after engraftment in a series of avian embryos. Remarkably,

Figure 1. Set up and characterization of melanoma cell lines and patient samples engrafted in the avian embryo. ▶

A Schematic diagram of the engrafting procedure of melanoma cell lines or melanoma biopsies in the chick embryo.

B 3D views (light-sheet imaging) of HH25 chick embryos engrafted with the A375P::GFP stable melanoma cell labeled with an anti-GFP antibody (in red) and an anti-HNK1 antibody (in white, migrating and early post-migrating NCCs).

C, D Immunolabeling of HH25 chick embryo sections 50 h post-engraftment of A375P::GFP cells, using an anti-GFP antibody (in green in C, in brown in D, A375P:GFP cells), an anti-HNK1 antibody (in white, migrating and early post-migrating NCCs), an anti-Ki67 antibody (in red in C, cycling cells), an anti-SOX10 antibody (in red in D, also stains chick endogenous NCCs) and an anti-MITF antibody (in yellow in D). Nuclei were stained with Hoechst (in blue). In (C), the upper left photo shows a grafted embryo prior to cryosection. In (D), right panels are enlargements of the lower left panel. Scale bar: 200 μm.

E Tumor take rate of 13 melanoma patient samples engrafted in a series of chick embryos, ranked according to their assigned Stage (I to IV).

F 3D views (light-sheet imaging) of HH25 chick embryos engrafted with different melanoma patient samples, showing different patterns of tumor cell localization 50 h post-engraftment.

G Immunofluorescent labeling of HH25 chick embryo sections 50 h post-engraftment of OF-MEL-020 patient sample, labeled with CFSE (in green) prior to the graft. An anti-SOX10 antibody (in red) and an anti-MITF antibody (in yellow) were used. Nuclei were stained with Hoechst (in blue). Right panels are enlargements of the dotted area in the left panel.

Data information: So: Somite; NT: Neural Tube; No: Notochord; DRG: Dorsal Root Ganglia; SG: Sympathetic Ganglia; Coe: Coelom; *: initial graft site localization.
Source data are available online for this figure.

 © 2023 The Authors

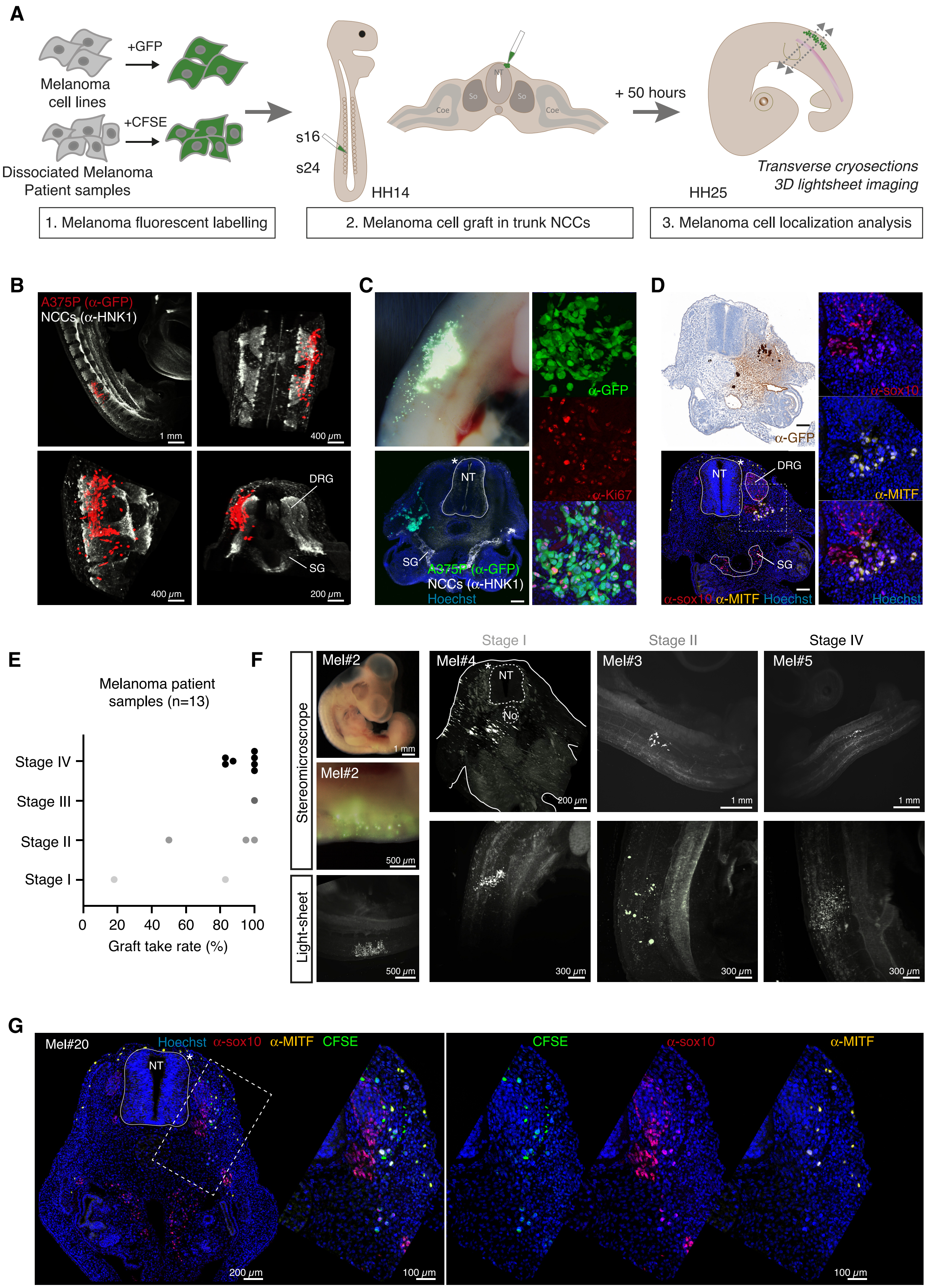
A
Melanoma cell lines
+GFP
+CFSE
Dissociated Melanoma Patient samples
s16
s24
HH14
+ 50 hours
HH25
Transverse cryosections
3D lightsheet imaging
1. Melanoma fluorescent labelling
2. Melanoma cell graft in trunk NCCs
3. Melanoma cell localization analysis
B
A375P (α-GFP)
NCCs (α-HNK1)
1 mm
400 μm
400 μm
DRG
SG
200 μm
C
α-GFP
α-Ki67
NT
SG
A375P (α-GFP)
NCCs (α-HNK1)
Hoechst
D
α-GFP
α-sox10
α-MITF
NT
DRG
SG
α-sox10 α-MITF Hoechst
Hoechst
E
Melanoma patient samples (n=13)
Stage IV
Stage III
Stage II
Stage I
0 20 40 60 80 100
Graft take rate (%)
F
Stereomicroscrope
Light-sheet
Mel#2
1 mm
500 μm
Stage I
Stage II
Stage IV
Mel#4
NT
No
200 μm
Mel#3
1 mm
Mel#5
300 μm
G
Mel#20
Hoechst α-sox10 α-MITF CFSE
NT
CFSE
α-sox10
α-MITF
200 μm
100 μm

Figure 1.

successful tumor graft was observed for each patient with a graft take rate above 50% for all patient samples, with the exception of the OF-MEL-001 sample showing a very low cellularity (Fig 1E and F). As for engrafted human TNBC samples (Jarrosson *et al*, 2021), the tumor take was not conditioned by tumor stage or proliferative index (nb of mitosis/mm^2) (Fig 1E and F and Table EV1).

Analysis of embryos by 3D light-sheet confocal microscopy revealed different distribution patterns of patient cells within the embryonic tissues, ranging from scattered cells with few cell-cell contacts (Mel#5, Fig 1F) to dense and cohesive tumor foci under the skin (Mel#4, Fig 1F). Notably, a given pattern was reproduced in all embryos engrafted with the same patient sample. We confirmed that a fraction of CFSE-labelled patient melanoma cells also expressed varying levels of MITF and/or SOX10 along their migration path, indicating that melanoma ITH was preserved after implantation (Fig 1G). The AVI-PDX^{TM} thus constitutes a highly efficient and reproducible approach to create melanoma PDX models.

Proliferative and invasive melanoma phenotypes are preserved in the avian xenograft model

We then assessed whether melanoma cell proliferative/invasive states (ITH), could be modeled in the AVI-PDX^{TM}. We analyzed the expression profiles of MITF and Ki67 in A375P-xenografts, in which dense tumor masses were surrounded by streams of migrating cells. Interestingly, the fraction of A375P cells showing low MITF expression was significantly higher in cells disconnected from tumor masses, suggesting a dynamic regulation of MITF expression depending on the proliferative (tumor masses) versus invasive (migrating cells) states of melanoma cells (Fig 2A and B). This was also reflected in the decrease of the fraction of Ki67-positive cells in the migrating population (Fig 2C and D) and in the mesenchymal morphology of Ki67-negative cells (Fig 2C).

Next, we investigated the possibility to mimic melanoma cell phenotypes and associated clinical responses to BRAFi/MEKi. For this, we engrafted A375R cells, a BRAFi resistant cell line generated *in vitro* upon chronic exposure of A375 to BRAFi, which has lost MITF while retaining SOX10 expression (Richard *et al*, 2016; Fig EV1B). As with A375P cells, MITF-SOX10 expression profile was maintained in A375R cells engrafted in the avian embryo (Fig EV1C and D). We also used a pair of BRAFi/MEKi sensitive/resistant primary cell lines, that we established from a BRAF^{V600}-mutated MM patient, before (GLO) (Richard *et al*, 2016) or after (GLO-R) the clinical emergence of resistance (Fig EV2A and B). MITF, SOX10 and ZEB2 expression levels were decreased in GLO-R cells while ZEB1 expression was increased (Fig EV2C), suggesting a phenotype switch upon acquisition of resistance. RNA-Seq analyses further highlighted an enrichment of a proliferative signature in GLO cells, while GLO-R cells were characterized by an invasive signature (Fig 2E).

We therefore assessed the behavior and distribution pattern of GLO and GLO-R cells in embryonic tissues. While GLO cells formed cohesive tumor masses immediately surrounded by isolated migrating cells, individual GLO-R cells were scattered under the skin and in deeper tissues, rarely forming clusters (Fig 2F). Quantification of tumor cell dispersion achieved by measuring the compaction index confirmed a significant difference between GLO and GLO-R tumor features (Fig 2G). Moreover, immunostainings showed that engrafted GLO cells maintained a high level of MITF and SOX10 expression compared to GLO-R cells, in which MITF expression was negligible (Fig EV2D). These data confirm that key molecular features of the SOX10-MITF axis in patient-derived primary cultures are preserved after grafting in the embryo. Thus, the divergent proliferative and invasive states of GLO and GLO-R cells are characterized by distinct distribution and phenotypic patterns within embryonic tissues. By recapitulating the embryonic microenvironment and preserving key intrinsic molecular features, the AVI-PDX^{TM} enables melanoma cells to translate their different SOX10-MITF levels into distinct migratory/invasive behaviors hence modeling ITH.

The AVI-PDX^{TM} robustly predicts drug efficacy in melanoma patients

We then investigated whether our AVI-PDX^{TM} could reproduce the heterogeneity of patient tumor responses to BRAFi/MEKi therapies.

Figure 2. Melanoma cells retain their phenotypic traits and associated response to targeted therapies in the AVI-PDX^{TM} model.

A, B Immunofluorescent labeling (A) of HH25 chick embryo sections 50 h post-engraftment of A375P::GFP cells, using an anti-GFP antibody, an anti-MITF antibody (pink) and an anti-SOX10 (white) antibody. Nuclei were stained with Hoechst. Right panels are enlargements of the dotted area in left panel. In (B), the mean fraction of MITF-negative (MITF^{neg}), -low (MITF^{low}) and -positive (MITF^{pos}) A375P::GFP cells in the tumor mass and in migrating cells was quantified (n = 16 slices from 3 embryos). Error bars show SEM. Mann-Whitney test comparing tumors *vs* migrating cells for each class of MITF expression was performed, P-values are indicated on the graph.

C, D Immunofluorescent labeling (C) of HH25 chick embryo sections 50 h post-engraftment of A375P::GFP cells, using an anti-GFP antibody and an anti-Ki67 antibody. Nuclei were stained with Hoechst. Right panels are enlargements of the dotted area in the left panel. In (D), the mean fraction of Ki67-positive (Ki67^{pos}) A375P::GFP cells in the tumor mass and in migrating cells was quantified (n = 25 slices from 3 embryos). Arrows point at cells having a mesenchymal morphology. Error bars show SEM. Student t-test, $^{**}P = 0.0070$.

E RNASeq analysis of GLO and GLO-R cells; ssGSEA Normalized Enrichment Score (NES) of gene signatures published in (Verfaillie *et al*, 2015), associated with either a proliferative or an invasive melanoma phenotype were scored in both cell lines. P-values are indicated in the graphical representation.

F, G 3D views (light-sheet imaging, F) of HH25 chick embryos engrafted with GLO or GLO-R cells labeled with CFSE (in green) prior to the graft. The total volume occupied by tumor cells and the number of segmented CFSE-positive objects was quantified in (G) to calculate a mean compaction index (see details in the Materials and Methods section) of tumors for each cell line. Each dot represents a tumor analyzed in a different embryo. Bars and error bars indicate mean + SEM. Mann-Whitney test, $P < 0.0001$.

H–K 3D views (H, J) and quantification of tumor volumes (I, K) of HH25 chick embryos engrafted with GLO (H, I) or GLO-R (J, K) cells and treated with a combination of Vemurafenib and Cobimetinib, or Vorinostat alone, or a combination of the three molecules. The number of embryos analyzed for each experimental condition are indicated on the graphs. Bars and error bars indicate mean + SEM. Student t-tests, $^{**}P < 0.01$, ns: not significant. Exact P-values are indicated on the graphs.

Source data are available online for this figure.

© 2023 The Authors

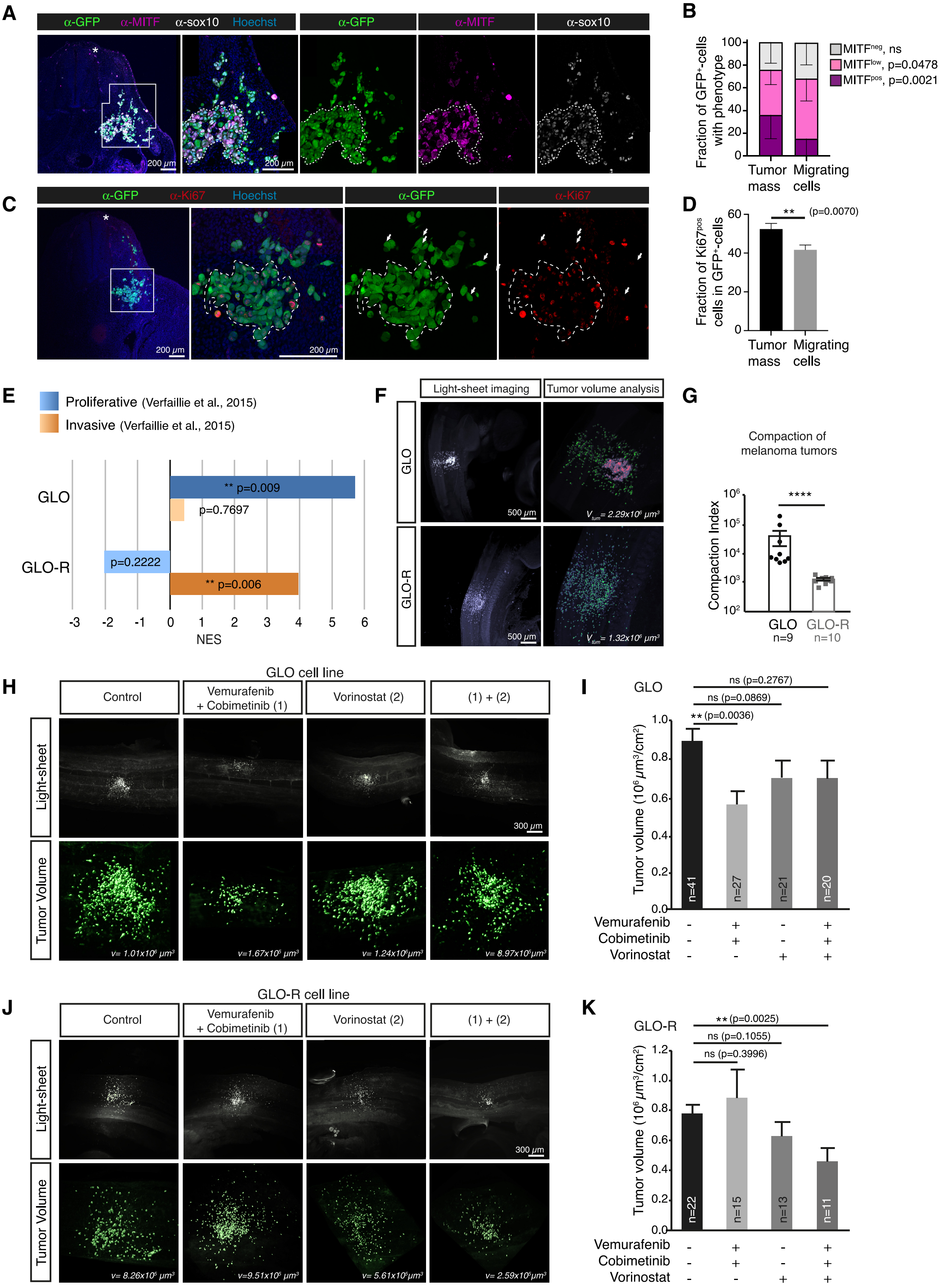
A
α-GFP α-MITF α-sox10 Hoechst
α-GFP
α-MITF
α-sox10
200 μm
B
Fraction of GFP+-cells with phenotype
MITFneg, ns
MITFlow, p=0.0478
MITFpos, p=0.0021
Tumor mass
Migrating cells
C
α-GFP α-Ki67 Hoechst
α-GFP
α-Ki67
D
Fraction of Ki67pos cells in GFP+-cells
** (p=0.0070)
E
Proliferative (Verfaillie et al., 2015)
Invasive (Verfaillie et al., 2015)
GLO
GLO-R
** p=0.009
p=0.7697
p=0.2222
** p=0.006
NES
F
Light-sheet imaging
Tumor volume analysis
500 μm
G
Compaction of melanoma tumors
Compaction Index
****
GLO n=9
GLO-R n=10
H
GLO cell line
Control
Vemurafenib + Cobimetinib (1)
Vorinostat (2)
(1) + (2)
Light-sheet
Tumor Volume
300 μm
I
GLO
ns (p=0.2767)
ns (p=0.0869)
** (p=0.0036)
Tumor volume (10^6 μm³/cm²)
n=41
n=27
n=21
n=20
Vemurafenib
Cobimetinib
Vorinostat
J
GLO-R cell line
K
GLO-R
** (p=0.0025)
ns (p=0.1055)
ns (p=0.3996)
n=22
n=15
n=13
n=11

Figure 2.

We further evaluated the efficacy of these therapies in combination with epigenetic drugs, namely HDAC (histone deacetylases) inhibitors such as Vorinostat, which emerged as promising therapeutic options to overcome resistance (Huijberts *et al*, 2020).

We determined the optimal dose of each therapeutic compound – that is, Vemurafenib, Cobimetinib, Vorinostat- in chick embryos by performing intravenous injections of increasing doses of each drug in chorioallantoic vessels of HH20 chick embryos (72 h post-fertilization) (Fig EV3A–C). Twenty-four hours post-injection, we quantified the survival rate, monitored the morphology and measured global embryonic growth by estimating the body surface area (BSA) of injected embryos, as described in the methods section and in previous work (Jarrosson *et al*, 2021). Survival rates below 75% or significant differences in BSA compared to the control group were indicative of dose toxicity, enabling to define the *in ovo* maximum tolerated dose (MTD) of Cobimetinib, Vemurafenib and Vorinostat (Fig EV3A–C). We then assessed the tumor growth of A375P/A375R, and GLO/GLO-R cells *in ovo* upon administration of Cobimetinib/Vemurafenib combination (Fig EV3D). Vemurafenib/Cobimetinib treatment triggered a significant reduction of A375P (Fig EV3E and F) and GLO (Fig 2H and I) tumor volumes as compared to control-treated embryos while A375R (Fig EV3E and F) and GLO-R (Fig 2J and K) tumor volumes were not affected. Consistently, an increase in the fraction of fragmented cells and a decrease in the fraction of dividing phospho-histone 3 positive cells was observed in A375P and GLO tumors upon treatment, while no significant difference was observed in A375R and GLO-R tumors (Fig EV3G–J). Hence, the sensitivity/resistance features of $BRAF^{V600}$-mutated melanoma cells to BRAFi/MEKi were maintained in the avian model.

In parallel, Vorinostat treatment was performed, either alone or in combination with BRAFi/MEKi in GLO and GLO-R cells (Fig 2H–K). Vorinostat alone did not impact GLO-tumor volumes and only triggered a slight decrease in GLO-R tumors. Conversely, co-injection of Vorinostat with Vemurafenib/Cobimetinib triggered a significant reduction of GLO-R tumor volumes, in agreement with recent data suggesting that BRAFi/MEKi resistance could, at least partially, be overcomed by epigenetic drugs (Wang *et al*, 2018; Fig 2J and K). Notably, when Vorinostat was combined with BRAFi/MEKi, the anti-tumor effect of BRAFi/MEKi on GLO-tumors was abrogated (Fig 2H and I). This observation corroborates previous studies suggesting that HDACi could antagonize BRAFi/MEKi activity in BRAFi/MEKi-sensitive melanoma cells (Wang *et al*, 2018). These findings suggest that our melanoma AVI-PDX^{TM} model may also be promising to evaluate drug efficacy in preclinical studies using mono- and combination therapies.

The AVI-PDX^{TM} allows relevant preclinical assessment of targeted therapies and is predictive of patient clinical response

We next evaluated the effect of BRAFi/MEKi treatment on patient samples with distinct mutational profiles after their implantation in chick embryos. A significant reduction in tumor volume was observed upon Vemurafenib/Cobimetinib treatment in OF-MEL-027 and OF-MEL-020 samples, both harboring a $BRAF^{V600E}$ mutation. Conversely, the $NRAS^{Q61L}$-mutated OF-MEL-028 sample, having a wild type *BRAF* status, did not show any significant response to Vemurafenib/Cobimetinib treatment *in ovo*, in accordance with its mutational status (Fig 3A and B).

We then studied whether the AVI-PDX^{TM} model could be predictive of the clinical response of patients. Three $BRAF^{V600E}$-mutated patients, treated with a Dabrafenib/Trametinib combination, were clinically scored at three months, which revealed three types of responses: stable disease (OF-MEL-033), partial response with local reduction of tumor foci (OF-MEL-034), or significant global response (OF-MEL-035). In parallel, replicas of these patient tumors were produced in avian embryos and treated with the same combination therapy, Dabrafenib and Trametinib, at their MTD (Fig 3C and D). Remarkably, not only did the clinical stable disease evaluation match the stable volume of tumors in the avian replicas (OF-MEL-033, Fig 3E and F), but the significant anti-tumor response to BRAFi/MEKi observed for patient OF-MEL-035 was also associated with a 46% decrease in tumor volume in avian replicas (Fig 3E and H). Moreover, the partial response of patient OF-MEL-034 was translated into a discrete mean tumor volume reduction, with a strong heterogeneity of response between the tumor replicas (Fig 3E and G). Thus, this analysis revealed a striking similarity between the clinical outcome of the patient and the short-term response of avian replicas.

Discussion

Our study depicts an alternative *in vivo* model of melanoma and provides an overview of its power to perform relevant preclinical studies. By implanting cell lines, patient-derived short-term cultures but also fresh or frozen patient biopsies at the level of the MSA in HH13 chick embryos, melanoma cells were driven to form tumors in the skin and under the epidermis, within 2 days. Interestingly, grafted cells followed typical endogenous melanoblast migrating routes to reach the developing dermis and epidermis. There, tumor cells maintained their phenotypic heterogeneity and plasticity regarding their proliferative and invasive properties, but also the expression of their corresponding key markers. Notably, tumor take was obtained for the 13 (100%) patient biopsies used in the study, with a tumor take rate above 80% for 11 samples (85%), irrespective of the stage, mitotic index, the metastatic/primary origin of the biopsy or the mutational status. The full sequence depicted here takes place between HH13 and HH25. At these early stages, chicken embryos show neither functional innate nor adaptive immunity. This may provide an immuno-permissive microenvironment for xenografts (Alkie *et al*, 2019; Dóra *et al*, 2017; Garcia *et al*, 2021) but precludes, in this set up, studies involving the host immune system.

Melanoma PDX models classically set up in immunocompromised mice are associated with major constraints among which the need for large amounts of tumor material incompatible with most melanoma biopsies, a low graft take efficiency (around 60% for skin melanomas; Krepler *et al*, 2017) and a long-term establishment (mean of 10 weeks to palpable masses) limiting statistical analyses and precluding studies designed for personalized medicine. Moreover, costs and ethical issues strongly limit mouse PDX applications. We report here a miniaturized *in vivo* model suited for melanoma patient biopsies, that can be implanted in a series of embryos without any culture step, leading to fast, efficient and reproducible graft take in a relevant microenvironment that models melanoma cell heterogeneity and associated resistant/sensitive

 © 2023 The Authors

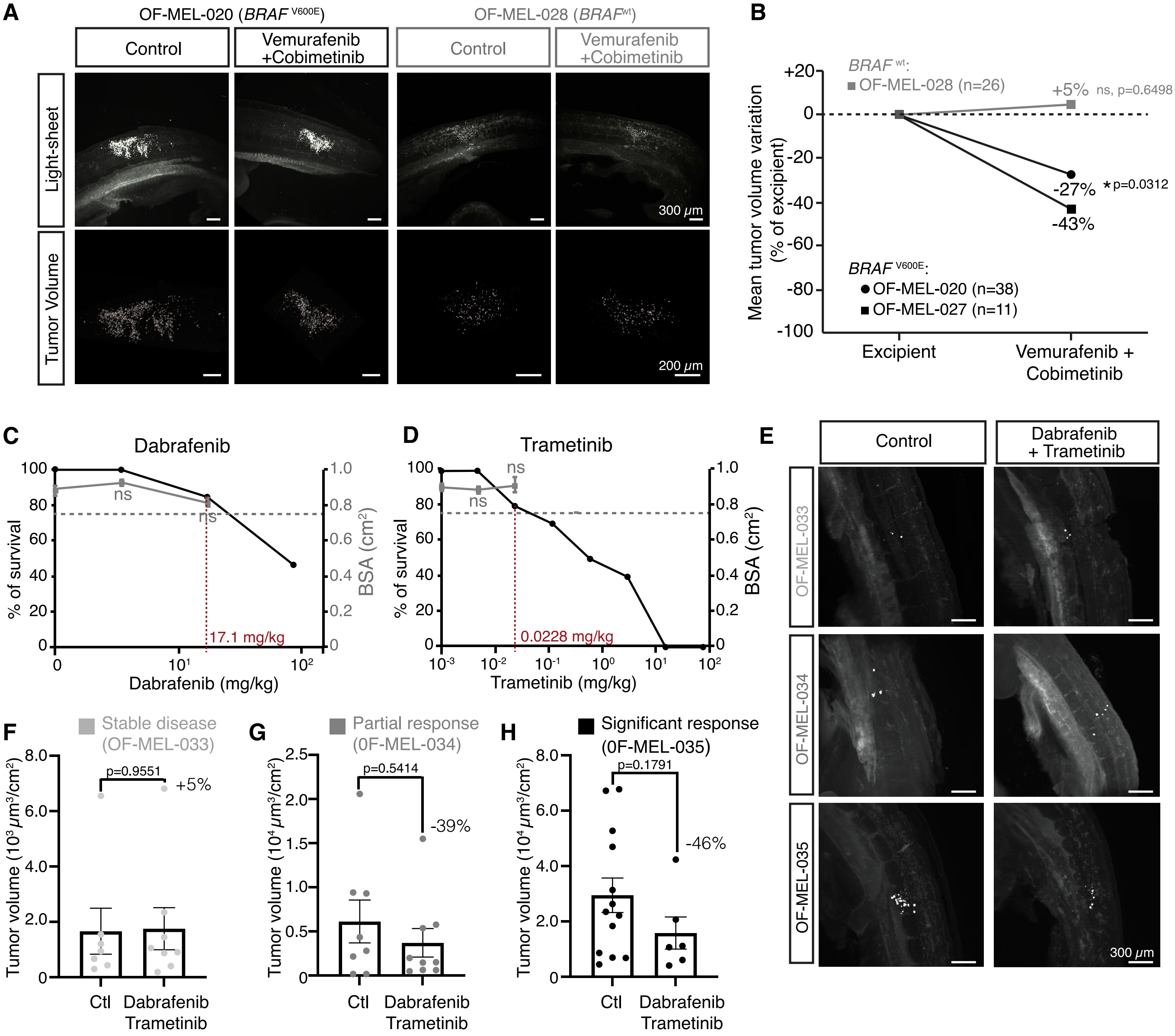

Figure 3. The AVI-PDX^{TM} paradigm efficiently models patient clinical response to targeted therapies.

A, B 3D views (A) and quantification of variations in the mean tumor volume (B) of HH25 chick embryos engrafted with BRAF^{wt} (OF-MEL-028) or BRAF^{V600E} (OF-MEL-020, OF-MEL-027) patient samples and treated with excipient or a combination of Vemurafenib and Cobimetinib. The number of embryos analyzed for each patient sample is indicated on the graphs. Student t-test for OF-MEL-020; Mann-Whintey test for OF-MEL-028, *$P < 0.05$, ns: not significant. Exact *P*-values are indicated on the graphs.

C, D Survival rate (left axis) and mean body surface area (BSA, right axis) of chick embryos injected with increasing doses of Dabrafenib (C) and Trametinib (D). The maximum tolerated dose (MTD, in red on the *X*-axis) was defined as the higher dose of drug associated with a survival rate higher than 75% and a mean BSA similar (i.e., non-statistically different) from embryos treated with excipient (17.1 and 0.0228 mg/kg respectively). $N = 10$ replicates per experimental group. Error bars indicate SEM. ns, non-significant using Student's *t*-test compared with excipient.

E–H 3D views (E) and quantification of tumor volumes (F–H) of HH25 chick embryos engrafted with 3 different patient samples (OF-MEL-033 [F], OF-MEL-034 [G], OF-MEL-035 [H]) and treated with excipient or a combination of Dabrafenib and Trametinib. The clinical response of each patient after a 3 months treatment with Dabrafenib/Trametinib is indicated above the graphs. Error bars indicate SEM. Mann-Whitney tests, exact *P*-values are indicated on the graphs.

Source data are available online for this figure.

profiles to targeted therapies. Lightsheet 3D imaging of the tumors implies that quantifications are made at endpoint only, precluding time frame analyses of tumor growth, which can rather be evaluated by measuring tumor volumes in independent, statistically relevant, experimental groups stopped at different time points. Some of these particularities are shared with melanoma xenografts performed in zebrafish embryos that provide a complementary, valuable and efficient alternative paradigm, especially suited for

high throughput drug screening (Yan *et al*, 2019; Das *et al*, 2020; Xiao *et al*, 2020).

The AVI-PDX™ brings novel solutions for studies based on patient samples and personalized medicine, which mainly rely on the successful engraftment of patient biopsies, the clinically relevant mode of therapy administration (iv) and the possibility to assess metastatic extension in specific organs. Of note, at developmental stages used herein, the use of chicken embryos is in perfect accordance with ethical guidelines according to the European directive 2010/63/EU. We have previously documented the possibility to harvest and sort viable human tumor cells from avian tissues days after the graft (Delloye-Bourgeois *et al*, 2017; Jarrosson *et al*, 2021). However, whether such post-graft material can be expanded and/or cryopreserved to perform serial engraftment in the avian embryo, although theoretically feasible, needs further experimental evaluation. The latter potential limitation is indeed circumvented with patient-derived organoids (PDO), that hold the advantage to be expandable and cryopreservable, allowing traceability and repeatability of preclinical experiments. While melanoma PDO may be too long to establish for personalized medicine purposes (Ronteix *et al*, 2022), using them as high-quality starting material to perform xenografts in animal models has proven efficient (Vilgelm *et al*, 2020) and would be a valuable approach to combine with the AVI-PDX™ *in vivo* paradigm and to optimize for preclinical studies.

Importantly, the results of our prospective analysis suggest that the response to targeted therapies injected in melanoma AVI-PDX™ are predictive of initial patient response to BRAFi/MEKi, within a timeframe compatible with therapeutic decision-making which is a key criterion of personalized medicine. In addition, we could model synergistic/antagonistic behaviors of combination of targeted therapies depending on melanoma cell phenotypes, underlining the power of our *in vivo* paradigm to provide relevant information on the efficacy of candidate molecules, even in complex therapeutic regimens. Longer treatments were not experienced in this study but are compatible with the AVI-PDX™ (Ben Amar *et al*, 2022) and could help addressing other questions, such as responses to sequential therapies or effects on metastatic extension. Overall, we provide proof-of-concept that the AVI-PDX™ model accurately reproduces melanoma patient response to BRAFi/MEKi, and is highly suited to assess the efficacy of drug combinations.

Immunotherapies targeting negative regulatory checkpoints on immune cells are another efficient therapeutic option for melanoma patients, altough 60% of patients still show resistance to anti-PD-1 blocking antibodies (Larkin *et al*, 2019). The most effective first-line treatment and the optimal sequencing of immune and targeted therapies remain to be determined. Some studies suggest lower activity of immunotherapy after BRAFi/MEKi treatment (Simeone & Ascierto, 2017; Amini-Adle *et al*, 2018). Mechanisms of cross-resistance between targeted and immunotherapies were recently characterized (Haas *et al*, 2021), but there are limited data on BRAFi/MEKi after immunotherapy failure (Xia *et al*, 2018; Rogala *et al*, 2022). Several clinical trials are ongoing to compare the efficacy of different sequences and regimens of BRAFi/MEKi therapy and immunotherapy. Further technological developments will be necessary to allow an accurate evaluation of immunotherapies in the AVI-PDX™.

For $BRAF^{V600}$-mutated MM patients, the clinical decision about whether to use first-line targeted therapy or immunotherapy is currently based on pluridisciplinary discussions considering medical history and clinical characteristics but biomarkers are lacking. There is thus an urgent medical need to define which $BRAF^{V600}$-mutated MM patients would benefit or not from a BRAFi/MEKi treatment or would better be directed towards first-line immunotherapy.

In the future, our study should pave the way for the design of a test of personalized medicine, thus improving the therapeutic decision-making process for $BRAF^{V600}$ melanoma patients.

Material and Methods

Anticancer drugs

Vemurafenib (PLX4032), Cobimetinib (GDC-0973), Vorinostat (SAHA), Dabrafenib (GSK2118436) and Trametinib (GSK1120212) were purchased from Selleckchem (stock solution at 10 mM). Those chemicals were diluted in DMSO-0.5% Tween 80 used as an excipient, for *in vivo* experiments.

Chick embryos

Embryonated eggs were obtained from a local supplier (Couvoir de Cerveloup, Vourey, France). Laying hen's sanitary status was regularly checked by the supplier according to French laws. Eggs were housed in an incubator at 18°C until further use. They were then incubated at 38.5°C in a humidified incubator until the desired developmental stage. In all experiments, embryos were randomized in each experimental group and were harvested at embryonic day 4 (4 days post-fertilization).

Cell lines

Cell lines were regularly tested for mycoplasma contamination over the duration of the experiments.

The A375P human melanoma cell line obtained from ATCC and cultured in DMEM supplemented with 10% FBS (Cambrex) and 100 U/ml penicillin-streptomycin (Invitrogen). Stable expression of GFP in A375P was obtained by transduction of HIV1-based lentiviral particles as explained below. Generation of A375R was previously described (Richard *et al*, 2016). A375R cells were cultured in media containing 3 μM of Vemurafenib.

Patient-derived short-term cultures (< 10) were established from a $BRAF^{V600}$ metastatic melanoma patient, before treatment for GLO, or after emergence of resistance to Vemurafenib/Cobimetinib for GLO-R. These short-term cell cultures were grown in RPMI complemented with 10% FBS and 100 U/ml penicillin-streptomycin.

Viral infection and plasmid

Self-inactivating HIV1-derived vector was produced by the lentivectors production facility/SFR BioSciences Gerland—Lyon Sud (UMS3444/US8) and encodes the green fluorescent protein (GFP) under the control of a SFFV promoter (SIN-HIV-SFFV-eGFP). Briefly A375P cells were plated in six well plates (5×10^5 cells per well) in complete medium. After 2 h medium was replaced with 2 ml

© 2023 The Authors

medium containing 2% FBS and 2 mg/ml polybrene (Sigma). After an hour this medium was removed and replaced with 2 ml of medium containing 5×10^6 IU of lentiviral vector. After 16 h medium was removed and cells rinsed and incubated with normal medium (10% FCS). Analysis by FACS showed that close to 100% of cells were positive for GFP. Medium from semi-confluent transduced cells showed no capacity to transfer GFP expression to naive control cell lines, indicating that infectious viruses were not produced by the transduced cells.

Human samples

All experiments involving human samples conformed to the principles set out in the WMA Declaration of Helsinki and the Department of Health and Human Services Belmont Report.

Patient samples OF-MEL-001, OF-MEL-002, OF-MEL-003, OF-MEL-004, OF-MEL-005, OF-MEL-025, OF-MEL-026, OF-MEL-027, OF-MEL-028 and associated histological and clinical data were obtained from the Biological Resource Center of the Lyon Sud Hospital (Hospices Civils de Lyon) following surgery as their standard of care with patient's informed signed consent to reuse biological samples for research purposes. Human melanoma sample OF-MEL-020 was obtained from NeuroBioTec (CRB HCL, Lyon France, Biobank BB-0033-00046) and is part of a collection registered at the French Department of Research (DC 2008-72).

Cutaneous melanoma biopsies OF-MEL-033, OF-MEL-034, OF-MEL-035 were obtained from patients included in the clinical trial NCT0439672, performed in the Lyon Sud Hospital (Hospices Civils de Lyon). Cutaneous biopsies were taken either from primary lesions or cutaneous metastases, and biopsied before treatment. Human tumor samples and clinical data were collected once the patients signed their informed consent to be included in the study. This minimally invasive study was approved by the national health authorities and ethics committee "Comité de Protection des Personnes Sud Méditerranée III" (n° ANSM 2019-A00900-57). Following surgery, resected tumors and biopsies were collected and stored in AqIX-RSI sterile medium (AqIX) for a maximum of 24 h. All samples were cryopreserved prior to engraftment, except for OF-MEL-001 and OF-MEL-026 samples which were directly implanted in avian embryos as fresh samples.

Human frozen samples

Tumors were washed with Ca^{2+},Mg^{2+}-free phosphate-buffered saline (PBS) (Life Technologies), crushed with a sterile scalpel into small tissue pieces of 1 mm^3, and put in freezing medium containing DMEM Glutamax medium (Gibco) supplemented with 10% FBS (Pan-biotech) and 10% DMSO (Sigma-Aldrich) in a cryotube prior to cryopreservation.

In ovo xenografts of melanoma samples

Embryonated eggs were incubated at 38.5°C in a humidified incubator until HH14 stage. Fresh or frozen patient samples were dissociated in Hank's Balanced Salt Solution (HBSS) with 156 units/ml of type IV collagenase, 200 mM CaCl2 and 50 units/ml DNase I for 20 min at 37°C and then incubated with 5 mg/ml trypsin for 2 min at 37°C under gentle mixing. Non-dissociated tissue was removed by filtration trough 0.4 μm nylon cell strainer (BD falcon). Non-fluorescent cell lines or patient samples were labeled with an 8 μM CFSE solution (Life Technologies). Stage HH13 chick embryos were grafted with fluorescent cells at the top of the dorsal neural tube within the migration staging area, with a glass capillary connected to a pneumatic PicoPump (PV820, World Precision Instruments) under a fluorescence stereomicroscope. For cell lines, approximately 2,500 living cells were grafted in each embryo, 200–300 for patient samples. For patient samples, the full cellular content obtained after dissociation was engrafted possibly including stromal and/or immune cells. Sample size (number of grafted embryos) for patient samples was not estimated as the total and limited amount of material available was used. For engraftment of cell lines, sample size was not estimated using statistical methods but was rather based on previous studies using avian embryos (Delloye-Bourgeois *et al*, 2017; Jarrosson *et al*, 2021; Ben Amar *et al*, 2022).

Drug administration and determination of drug maximum tolerated dose in chick embryos

For the determination of drug maximum tolerated dose, drugs were injected intravenously in chorioallantoic vessels. Twenty-four hours after injection, chick embryos were harvested, weighed (Sartorius Quintix35-1S) and measured along the rostro-caudal axis using the Leica LASX image analysis software. The Body Surface Area (BSA) was calculated using Dubois & Dubois formula: BSA (m^2) = 0.20247 × height (m)$^{0.725}$ × weight (kg)$^{0.425}$. The morphology/anatomy of each embryo was systematically analyzed to check their correct stage-related development. The criteria observed were: the survival (heart beating), the craniofacial morphology (presence of each cerebral compartment and eyes), the presence of four limb buds, the cardiac morphology, and the anatomy of embryonic annexes such as the allantois.

For evaluation of drug effect on xenografts, drugs were injected 24 h after melanoma cells graft. Prior to treatment, living grafted embryos were randomized into experimental groups and allocated to treatment after randomization.

According to pre-established criteria, dead embryos or embryos showing aberrant morphogenetic/growth criteria were excluded from the analysis.

Immunofluorescence on cryosections

Chick embryos were harvested and fixed in 4% Paraformaldehyde (PFA). Embryos were embedded in 7.5% gelatin—15% sucrose in PBS to perform 20 μm transverse cryosections. Heat-induced epitope retrieval was performed by immersion in antigen unmasking solution (citrate buffer) at 70°C for 2 h. Permeabilization and saturation of sections were performed in PBS—3% Bovine Serum Albumin (BSA)—0.5%. Triton. Anti-Ki67 (1/200, ab15580, Abcam, RRID:AB_443209), anti-MITF (1/100, clone C5, MAB3747, Merck-millipore, RRID:AB_570596), anti-HNK1 (1/50, clone 3H5, DSHB), anti-SOX10 (1/200, 89356, Cell Signaling, RRID:AB_2792980) and anti-phospho Histone H3 (1/500, MABE76, Millipore/Sigma, RRID:AB_11205074) were applied to cryosections and incubated overnight at 4°C. Alexa 555 anti-rabbit IgG (1/500, A21429, Thermo-Fisher Scientific, RRID:AB_2535850), Alexa 555 anti-mouse IgG (1/

500, A31570, ThermoFisher Scientific, RRID:AB_2536180), FluoProbes 647H donkey anti-mouse IgG (1/500, FPSC4110, Interchim), FP547H anti-rat (1/500, FPSB61110, Interchim) were used as secondary antibody. Nuclei were stained with Hoechst (H21486, Invitrogen). Slices were imaged with a confocal microscope (Olympus, FV1000, X81) using either a 10× objective for whole slice imaging or a 40× objective to focus on Ki67, MITF and SOX10 immunolabeling.

Immunofluorescence analyses on paraffin-embedded samples

3-μm tissue sections were cut from PFA-fixed paraffin-embedded embryos. The sections underwent immunofluorescence staining using the OPAL™ technology (Akoya Biosciences) on a Leica Bond RX. A 7-color panel was designed. Anti-SOX10 (1/1000, Santa Cruz sc-365692, RRID:AB_10844002) and ant-MITF (1/200, Sigma, 284M-96, RRID:AB_1516912) primary antibodies were used. DAPI was used for nuclei detection. Sections were digitized with a Vectra Polaris scanner (Perkin Elmer, USA).

Tissue clearing, whole mount SPIM imaging and image analysis

PFA-fixed HH25 embryos were cleared using an adapted Ethyl-Cinnamate protocol (Klingberg *et al*, 2017). Briefly, tissues were dehydrated in successive ethanol baths finally cleared in Ethyl Cinnamate (Sigma, 112372). Cleared samples were imaged using the UltraMicroscope SPIM (LaVision Biotech). 3D-images were built using Imaris™ software. All image quantifications were performed in blind regarding the treatment. Volumetric analysis was performed using the Imaris™ "Surface" module adjusted on CFSE or GFP fluorescence. The compaction index was determined as the ratio between the total volume occupied by tumor cells in an engrafted embryo and the number of fluorescent ($CFSE^+$) objects segmented with the Imaris™ Surface module.

In vitro cell survival assays

The CellTiter-Glo Luminescent Cell Viability Assay (ATP assay) (Promega) was used. 1,000 cells in 96-well plates were treated with three by 3-fold dilutions of the indicated drugs (BRAFi PLX4032, MEKi GDC-0973) for 72 h in a final volume of 100 μL. Luminescence was measured (Tekan). Control wells with DMSO were used for normalization.

Immunoblot analyses

Cells were washed twice with PBS containing $CaCl_2$ and then lysed in a 100 mM NaCl, 1% NP40, 0.1% SDS, 50 mM Tris pH 8.0 RIPA buffer supplemented with a complete protease inhibitor cocktail (Roche, Mannheim, Germany) and phosphatase inhibitors (Sigma-Aldrich). Protein expression was examined by Western blot using the anti-ZEB1 (1/200, Sigma HPA027524, RRID:AB_1844977), anti-ZEB2 (1/500, Sigma HPA003456, RRID:AB_10603840), anti-MITF (clone C5, ab80651, 1/500, Abcam, RRID:AB_1603129), anti-SOX10 (Santa Cruz, sc-365692, RRID:AB_10844002) antibodies for primary detection. Loading was controlled using the anti-GAPDH (1/20,000, Millipore) antibody. Horseradish peroxidase-conjugated rabbit anti-mouse, goat anti-rabbit, and donkey anti-goat polyclonal antibodies (Dako, Glostrup, Denmark) were used as secondary antibodies. Western blot detections were conducted using the Luminol reagent (Santa Cruz).

The paper explained

Problem

Metastatic melanoma patients carrying a $BRAF^{V600}$ mutation can be treated with targeted therapies (BRAF and MEK inhibitors) but resistance occurs. Predicting patient response to targeted therapies is crucial to guide clinical decision, since these patients may also be directed to first-line immunotherapy. Mouse patient-derived xenograft (PDX) models are incompatible with personalized medicine approaches because of their long timeframe.

Results

Herein, we developed a highly efficient patient-derived xenograft model using the avian embryo as a host (AVI-PDX™), enabling fast (few days) and reproducible tumor engraftment of melanoma patient samples, preserving key molecular and phenotypic features. We show that response to targeted therapies can be reliably modeled in these AVI-PDX™, making it a valuable preclinical tool for assessing efficacy of combination treatments in melanoma.

Impact

We provide proof-of-concept that the AVI-PDX™ models the diversity of responses of melanoma patients to BRAFi/MEKi, within days, hence positioning it as a valuable tool for the design of personalized medicine assays.

RNA-Seq

mARN from GLO and GLO-R were extracted in duplicates with the RNeasy mini kit (Qiagen) with DNase treatment. RNA libraries were prepared with the NextFlex Rapid Directional mRNA-Seq kit (Bioo-Scientific) with polyA+ mRNA enrichment, and sequenced on the ProfileXpert platform, on an Illumina Nextseq500 sequencing machine with a single read protocol (75 bp; 30 M reads). After demultiplexing and trimming, trimmed reads were mapped using TopHat 2.1.00b (Trapnell *et al*, 2009) against Human genome (hg19, GRCh37 Feb. 2009 from UCSC) in order to identify expressed genes. Reads mapping on each transcript were numbered and normalized using Cufflinks v.2.1.1 (Trapnell *et al*, 2010), fold change between the different groups were calculated using median of groups, and P-values were calculated using a t-test with equal variance and no P-value correction. Those calculations were performed using a proprietary R script. Mapped reads for each sample were counted and normalized using FPKM method (Fragments Per Kilobase of exon per Milion of mapped reads). Differentially expressed transcripts ($|lFC| > 1.5$; $P < 0.05$) were analyzed between GLO versus GLO-R. Single sample GSEA (ssGSEA) scores were computed on FPKM normalized data through gsva R package.

Quantification and statistical analysis

Statistical treatment of data was performed with Prism 9.0e (GraphPad). Both normality (D'Agostino & Pearson test) and variance homoscedasticity (F test) were checked. In cases where experimental groups did not pass normality test, non-parametric tests were used. In cases where experimental groups passed normality

© 2023 The Authors

tests but had significantly different variances, corrections were applied (Welch's correction for *t*-test). All statistical tests were two-sided. The exact test and *P*-values are mentioned in the figure legends and in the figure respectively.

All experiments were performed at least three times in laboratory, except for graft experiments with patient biopsies where a given sample could be implanted in series of embryos only once.

Data availability

The data reported in this paper are deposited in the Gene Expression Omnibus (GEO) database under the accession number GSE206689.

Expanded View for this article is available online.

Acknowledgements
The authors would like to thank Séverine Croze and Joël Lachuer, from « ProfileXpert » (service de « Génomique & Microgénomique », Université Lyon 1, SFR santé LYON-EST, UCBL-INSERM US 7-CNRS UMS 3453) for RNA-Seq experiments. Nicolas Gadot and Clémentine Lenevé (PAR) for slides stainings. We also thank B. Manship for English editing. Y.T. was supported by a fellowship from Ligue Nationale contre le Cancer and M.P. by a fellowship from "Région Rhônes Alpes" and the Association pour la Recherche contre le Cancer (ARC). Oncofactory was supported by the program "Innovation clinique" of the Hospices Civils de Lyon-Lyonbiopôle. This work was conducted within the framework of the Labex DevWeCAN of Université de Lyon, within the program 'Investissements d'Avenir' (ANR-11-IDEX-0007) operated by the French National Research Agency (ANR) (V.C. and C.D.-B.), and with the support of Institut Convergence Plascan (Grant Number ANR-17-CONV-0002) (J.C and S.D.).

Author contributions
Loraine Jarrosson: Data curation; formal analysis; investigation; methodology. **Stephane Dalle:** Resources; data curation; project administration. **Clélia Costechareyre:** Data curation; formal analysis; investigation; methodology. **Yaqi Tang:** Formal analysis; methodology. **Maxime Grimont:** Investigation; methodology. **Maud Plaschka:** Formal analysis; methodology. **Marjorie Lacourrège:** Methodology. **Romain Teinturier:** Resources; formal analysis; writing—review and editing. **Myrtille Le Bouar:** Resources; project administration. **Delphine Maucort-Boulch:** Formal analysis; project administration. **Anaïs Eberhardt:** Resources; writing—review and editing. **Valérie Castellani:** Conceptualization; funding acquisition; writing—review and editing. **Julie Caramel:** Conceptualization; data curation; formal analysis; supervision; funding acquisition; investigation; writing—original draft; writing—review and editing. **Céline Delloye-Bourgeois:** Conceptualization; data curation; formal analysis; supervision; funding acquisition; investigation; writing—original draft; writing—review and editing.

Disclosure and competing interests statement
V.C. and C.D.-B. are co-founders of OncoFactory SAS (www.oncofactory.com). L.J., C.C., M.L. and R.T. are employees of OncoFactory SAS.

References

Alkie TN, Yitbarek A, Hodgins DC, Kulkarni RR, Taha-Abdelaziz K, Sharif S (2019) Development of innate immunity in chicken embryos and newly hatched chicks: a disease control perspective. *Avian Pathol* 48: 288–310

Amini-Adle M, Khanafer N, Le-Bouar M, Duru G, Dalle S, Thomas L (2018) Ineffective anti PD-1 therapy after BRAF inhibitor failure in advanced melanoma. *BMC Cancer* 18: 705

Ben Amar D, Thoinet K, Villalard B, Imbaud O, Costechareyre C, Jarrosson L, Reynaud F, Novion Ducassou J, Couté Y, Brunet J-F *et al* (2022) Environmental cues from neural crest derivatives act as metastatic triggers in an embryonic neuroblastoma model. *Nat Commun* 13: 2549

Caramel J, Papadogeorgakis E, Hill L, Browne GJ, Richard G, Wierinckx A, Saldanha G, Osborne J, Hutchinson P, Tse G *et al* (2013) A switch in the expression of embryonic EMT-inducers drives the development of malignant melanoma. *Cancer Cell* 24: 466–480

Das I, Gad H, Bräutigam L, Pudelko L, Tuominen R, Höiom V, Almlöf I, Rajagopal V, Hansson J, Helleday T *et al* (2020) AXL and CAV-1 play a role for MTH1 inhibitor TH1579 sensitivity in cutaneous malignant melanoma. *Cell Death Differ* 27: 2081–2098

Delloye-Bourgeois C, Bertin L, Thoinet K, Jarrosson L, Kindbeiter K, Buffet T, Tauszig-Delamasure S, Bozon M, Marabelle A, Combaret V *et al* (2017) Microenvironment-driven shift of cohesion/detachment balance within tumors induces a switch toward metastasis in neuroblastoma. *Cancer Cell* 32: 427–443.e8

Dóra D, Fejszák N, Goldstein AM, Minkó K, Nagy N (2017) Ontogeny of ramified CD45 cells in chicken embryo and their contribution to bursal secretory dendritic cells. *Cell Tissue Res* 368: 353–370

Dummer R, Ascierto PA, Nathan P, Robert C, Schadendorf D (2020) Rationale for immune checkpoint inhibitors plus targeted therapy in metastatic melanoma: a review. *JAMA Oncol* 6: 1957–1966

Garcia P, Wang Y, Viallet J, Macek Jilkova Z (2021) The chicken embryo model: a novel and relevant model for immune-based studies. *Front Immunol* 12: 791081

Goding CR, Arnheiter H (2019) MITF-the first 25 years. *Genes Dev* 33: 983–1007

Haas L, Elewaut A, Gerard CL, Umkehrer C, Leiendecker L, Pedersen M, Krecioch I, Hoffmann D, Novatchkova M, Kuttke M *et al* (2021) Acquired resistance to anti-MAPK targeted therapy confers an immune-evasive tumor microenvironment and cross-resistance to immunotherapy in melanoma. *Nat Cancer* 2: 693–708

Herrscher H, Robert C (2020) Immune checkpoint inhibitors in melanoma in the metastatic, neoadjuvant, and adjuvant setting. *Curr Opin Oncol* 32: 106–113

Hoek KS, Eichhoff OM, Schlegel NC, Döbbeling U, Kobert N, Schaerer L, Hemmi S, Dummer R (2008) In vivo switching of human melanoma cells between proliferative and invasive states. *Cancer Res* 68: 650–656

Hugo W, Zaretsky JM, Sun L, Song C, Moreno BH, Hu-Lieskovan S, Berent-Maoz B, Pang J, Chmielowski B, Cherry G *et al* (2016) Genomic and transcriptomic features of response to Anti-PD-1 therapy in metastatic melanoma. *Cell* 165: 35–44

Huijberts S, Wang L, de Oliveira RL, Rosing H, Nuijen B, Beijnen J, Bernards R, Schellens J, Wilgenhof S (2020) Vorinostat in patients with resistant BRAFV600E mutated advanced melanoma: a proof of concept study. *Future Oncol* 16: 619–629

Jarrosson L, Costechareyre C, Gallix G, Ciré S, Gay F, Imbaud O, Teinturier R, Marangoni E, Aguéra K, Delloye-Bourgeois C *et al* (2021) An avian embryo patient-derived xenograft model for preclinical studies of human breast cancers. *iScience* 24: 103423

Klingberg A, Hasenberg A, Ludwig-Portugall I, Medyukhina A, Männ L, Brenzel A, Engel DR, Figge MT, Kurts C, Gunzer M (2017) Fully automated evaluation of total glomerular number and capillary tuft size in nephritic kidneys using lightsheet microscopy. *J Am Soc Nephrol* 28: 452–459

© 2023 The Authors

Krepler C, Sproesser K, Brafford P, Beqiri M, Garman B, Xiao M, Shannan B, Watters A, Perego M, Zhang G *et al* (2017) A comprehensive patient-derived xenograft collection representing the heterogeneity of melanoma. *Cell Rep* 21: 1953–1967

Kulesa PM, Kasemeier-Kulesa JC, Teddy JM, Margaryan NV, Seftor EA, Seftor RE, Hendrix MJ (2006) Reprogramming metastatic melanoma cells to assume a neural crest cell-like phenotype in an embryonic microenvironment. *Proc Natl Acad Sci USA* 103: 3752–3757

Larkin J, Ascierto PA, Dréno B, Atkinson V, Liszkay G, Maio M, Mandalà M, Demidov L, Stroyakovskiy D, Thomas L *et al* (2014) Combined vemurafenib and cobimetinib in BRAF-mutated melanoma. *N Engl J Med* 371: 1867–1876

Larkin J, Chiarion-Sileni V, Gonzalez R, Grob J-J, Rutkowski P, Lao CD, Cowey CL, Schadendorf D, Wagstaff J, Dummer R *et al* (2019) Five-year survival with combined nivolumab and ipilimumab in advanced melanoma. *N Engl J Med* 381: 1535–1546

Marine J-C, Dawson S-J, Dawson MA (2020) Non-genetic mechanisms of therapeutic resistance in cancer. *Nat Rev Cancer* 20: 743–756

Mort RL, Jackson IJ, Patton EE (2015) The melanocyte lineage in development and disease. *Development* 142: 620–632

Patton E, Mueller K, Adams D, Anandasabapathy N, Aplin A, Bertolotto C, Bosenberg M, Ceol C, Burd C, Chi P *et al* (2021) Melanoma models for the next generation of therapies. *Cancer Cell* 39: 610–631

Rambow F, Rogiers A, Marin-Bejar O, Aibar S, Femel J, Dewaele M, Karras P, Brown D, Chang YH, Debiec-Rychter M *et al* (2018) Toward minimal residual disease-directed therapy in melanoma. *Cell* 174: 843–855.e19

Rambow F, Marine J-C, Goding CR (2019) Melanoma plasticity and phenotypic diversity: therapeutic barriers and opportunities. *Genes Dev* 33: 1295–1318

Richard G, Dalle S, Monet M-A, Ligier M, Boespflug A, Pommier RM, de la Fouchardière A, Perier-Muzet M, Depaepe L, Barnault R *et al* (2016) ZEB1-mediated melanoma cell plasticity enhances resistance to MAPK inhibitors. *EMBO Mol Med* 8: 1143–1161

Rogala P, Czarnecka AM, Cybulska-Stopa B, Ostaszewski K, Piejko K, Ziętek M, Dziura R, Rutkowska E, Galus Ł, Kempa-Kamińska N *et al* (2022) Long-term outcomes of targeted therapy after first-line immunotherapy in BRAF-mutated advanced cutaneous melanoma patients-real-world evidence. *J Clin Med* 11: 2239

Ronteix G, Jain S, Angely C, Cazaux M, Khazen R, Bousso P, Baroud CN (2022) High resolution microfluidic assay and probabilistic modeling reveal cooperation between T cells in tumor killing. *Nat Commun* 13: 3111

Simeone E, Ascierto PA (2017) Anti-PD-1 and PD-L1 antibodies in metastatic melanoma. *Melanoma Manag* 4: 175–178

Tang Y, Durand S, Dalle S, Caramel J (2020) EMT-inducing transcription factors, drivers of melanoma phenotype switching, and resistance to treatment. *Cancers (Basel)* 12: 2154

Trapnell C, Pachter L, Salzberg SL (2009) TopHat: discovering splice junctions with RNA-Seq. *Bioinformatics* 25: 1105–1111

Trapnell C, Williams BA, Pertea G, Mortazavi A, Kwan G, van Baren MJ, Salzberg SL, Wold BJ, Pachter L (2010) Transcript assembly and quantification by RNA-Seq reveals unannotated transcripts and isoform switching during cell differentiation. *Nat Biotechnol* 28: 511–515

Verfaillie A, Imrichova H, Atak ZK, Dewaele M, Rambow F, Hulselmans G, Christiaens V, Svetlichnyy D, Luciani F, Van den Mooter L *et al* (2015) Decoding the regulatory landscape of melanoma reveals TEADS as regulators of the invasive cell state. *Nat Commun* 6: 6683

Vilgelm AE, Bergdorf K, Wolf M, Bharti V, Shattuck-Brandt R, Blevins A, Jones C, Phifer C, Lee M, Lowe C *et al* (2020) Fine-needle aspiration-based patient-derived cancer organoids. *iScience* 23: 101408

Voabil P, de Bruijn M, Roelofsen LM, Hendriks SH, Brokamp S, van den Braber M, Broeks A, Sanders J, Herzig P, Zippelius A *et al* (2021) An ex vivo tumor fragment platform to dissect response to PD-1 blockade in cancer. *Nat Med* 27: 1250–1261

Wang L, Leite de Oliveira R, Huijberts S, Bosdriesz E, Pencheva N, Brunen D, Bosma A, Song J-Y, Zevenhoven J, Los-de Vries GT *et al* (2018) An acquired vulnerability of drug-resistant melanoma with therapeutic potential. *Cell* 173: 1413–1425.e14

Wouters J, Kalender-Atak Z, Minnoye L, Spanier KI, De Waegeneer M, Bravo González-Blas C, Mauduit D, Davie K, Hulselmans G, Najem A *et al* (2020) Robust gene expression programs underlie recurrent cell states and phenotype switching in melanoma. *Nat Cell Biol* 22: 986–998

Xia CY, Wang DY, Mason R, Smith JL, McKean MA, Lo S, Guminski AD, Long GV, Carlino MS, Atkinson V *et al* (2018) Activity of targeted therapy after failure of first-line immunotherapy in BRAF-mutant metastatic melanoma. *J Clin Oncol* 36: 9532

Xiao J, Glasgow E, Agarwal S (2020) Zebrafish xenografts for drug discovery and personalized medicine. *Trends Cancer* 6: 569–579

Yan C, Brunson DC, Tang Q, Do D, Iftimia NA, Moore JC, Hayes MN, Welker AM, Garcia EG, Dubash TD *et al* (2019) Visualizing engrafted human cancer and therapy responses in immunodeficient zebrafish. *Cell* 177: 1903–1914.e14

License: This is an open access article under the terms of the Creative Commons Attribution License, which permits use, distribution and reproduction in any medium, provided the original work is properly cited.

Expanded View Figures

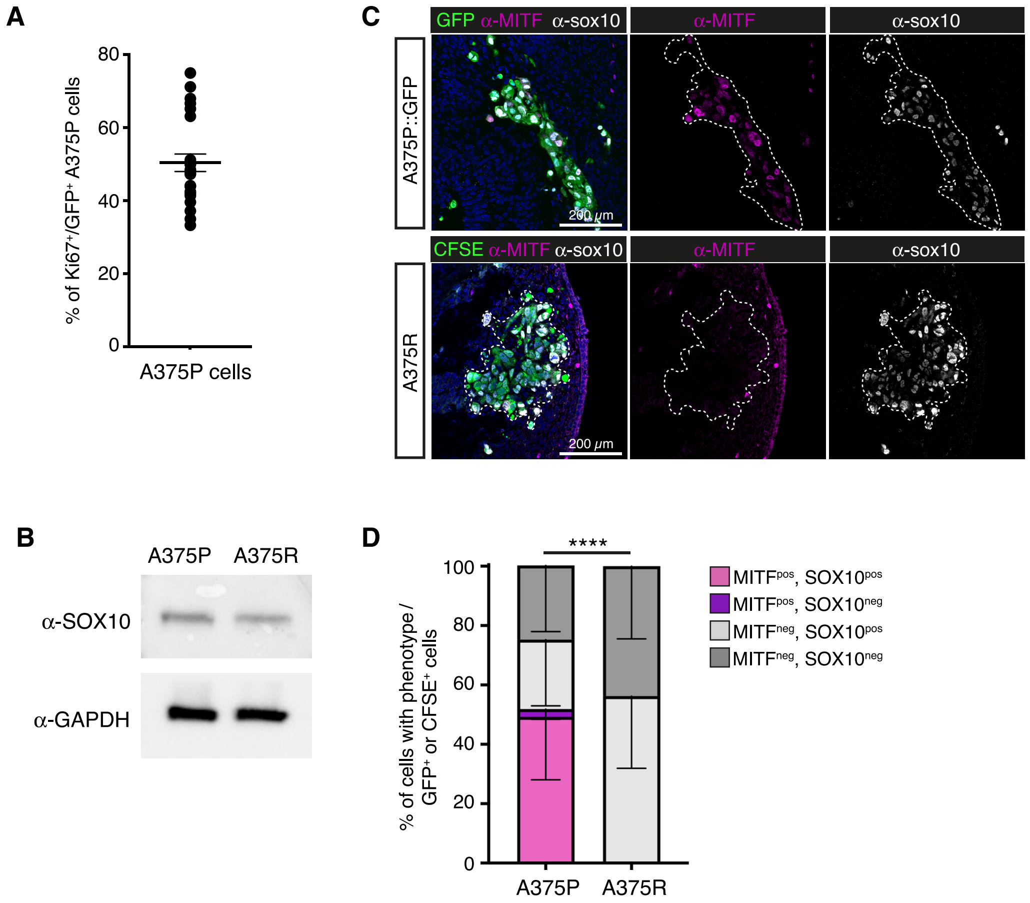

Figure EV1. Characterization of A375P and A375R cell lines in the avian graft model.

A Quantification of Ki67 immunofluorescent staining in GFP^+-A375P cells, 48 h after their graft in avian embryos (related to Fig 1C) ($n = 24$ sections from $N = 3$ embryos). Bars and error bars indicate mean ± SEM.

B Detection of SOX10 expression by Western blot in A375P and A375R cells, using GAPDH as a loading control.

C Immunofluorescent labelling of MITF and SOX10 in HH25 avian embryos engrafted with A375P::GFP cells or A375R cells labelled with CFSE prior to the graft.

D Quantification of the fraction of A375P (GFP^+, $n = 23$ sections from $N = 3$ embryos) and A375R ($CFSE^+$, $n = 21$ sections from $N = 3$ embryos) cells showing a positive or negative staining for SOX10 and/or MITF. Error bars indicate SEM. ****$P < 0.0001$, using Chi-square test comparing proportions of phenotypes in A375P versus A375R cell lines.

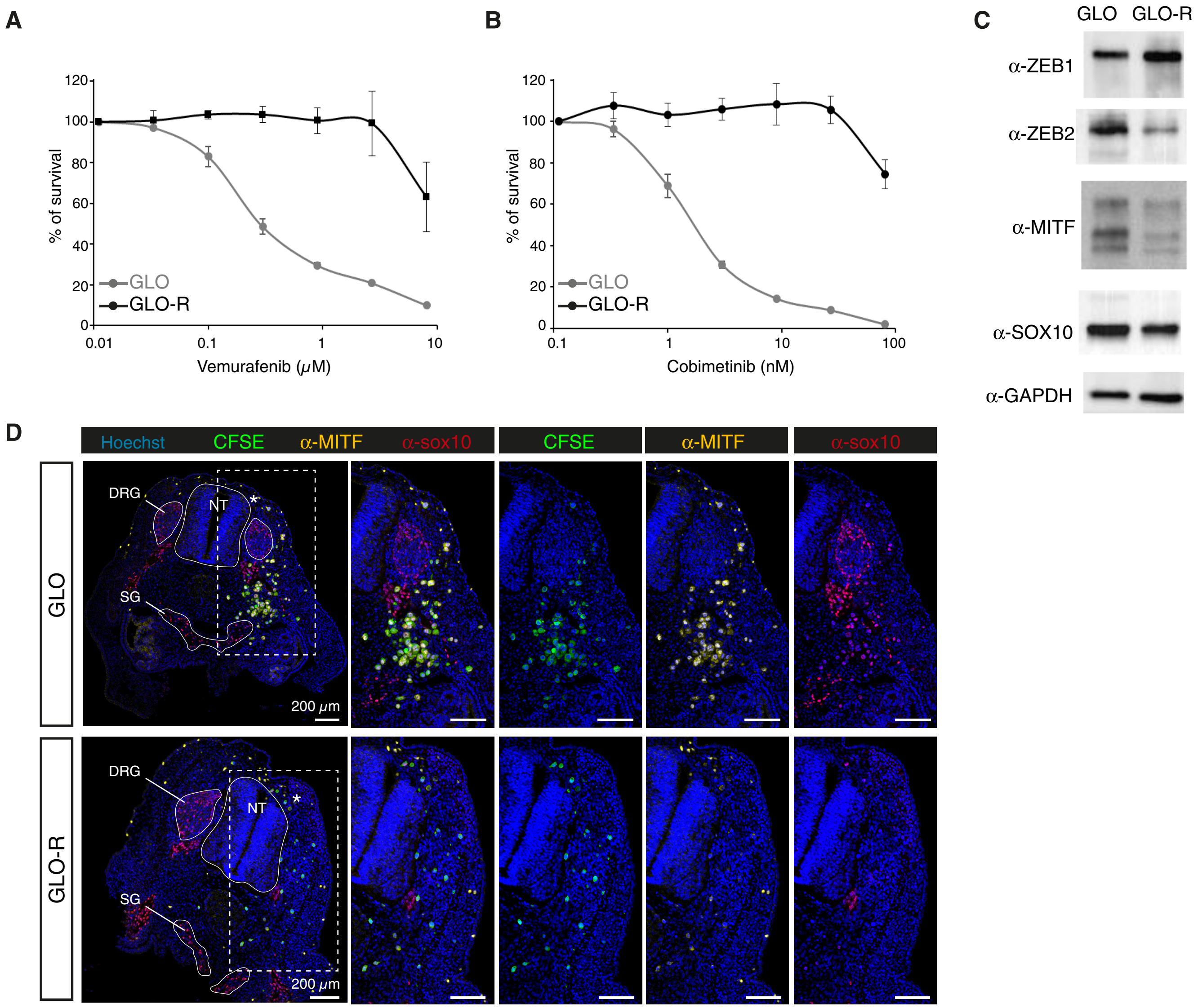

Figure EV2. Characterization of GLO and GLO-R cell lines *in vitro* and in the AVI-PDX^{TM} model.

A, B Survival rate of GLO and GLO-R cells upon exposure to increasing doses of Vemurafenib (A) or Cobimetinib (B) for 72 h. (n = 3 technical replicates for each cell line; two biological replicates of the full experiment have been performed). Error bars show SEM.

C Detection of ZEB1, ZEB2, MITF and SOX10 expression by Western blot in GLO and GLO-R cells, using GAPDH as a loading control.

D Immunofluorescent labelling of MITF (yellow) and SOX10 (red) in HH25 avian embryos engrafted with GLO or GLO-R cells, labelled with CFSE prior to the graft. Right panels are enlargements of the left panel for GLO and GLO-R grafts.

Data information: NT: Neural Tube; DRG: Dorsal Root Ganglia; SG: Sympathetic Ganglia.

 © 2023 The Authors

Figure EV3. Effect of Vemurafenib and Cobimetinib on melanoma cells engrafted in avian embryos.

A–C Survival rate (left axis) and mean body surface area (BSA, right axis) of avian embryos injected with increasing doses of Vemurafenib (A), Cobimetinib (B) or Vorinostat (C). Each dose was administered to a minimum of 10 embryos, using excipient (NaCl) as a control. The maximum tolerated dose (MTD) was defined as the higher dose of drug associated with a survival rate higher than 75% and a mean BSA similar (i.e., non-statistically different) from embryos treated with NaCl. MTDs are indicated in red on the abscissa axis.

D Schematic diagram of the grafting procedure followed by therapy administration and assessment of therapy effect on implanted melanoma cells/biopsies.

E, F 3D views (D) and quantification of tumor volumes (G) of HH25 chick embryos engrafted with A375P or A375R cells and treated with a combination of Vemurafenib and Cobimetinib or with excipient. Scale bar: 300 μm. The numbers of embryos analyzed are indicated on the graphs.

G, H Immunostaining (G) and quantification (H) of Vemurafenib/Cobimetinib co-administration effect on avian grafts of A375P and A375R cells. Dividing cells are labeled with anti-phospho Histone 3 antibody (a-pH3) and are highlighted with red arrows. The numbers of sections analyzed are indicated on the graphs are were obtained from at least three embryos per condition.

I, J Immunostaining (I) and quantification (J) of Vemurafenib/Cobimetinib co-administration effect on avian grafts GLO and GLO-R cells. Green arrows point at dying cells with dense cytoplasmic bodies. Dividing cells are labelled with anti-phospho Histone 3 antibody (α-pH3) and are highlighted with red arrows. Scale bar: 100 μm. The numbers of sections analyzed are indicated on the graphs are were obtained from at least three embryos per condition.

Data information: Error bars indicate SEM. $^{**}P < 0.01$, $^{****}P < 0.0001$, ns, non-significant using Student's *t* test pr Mann-Whintey test comparing excipient versus Vemurafenib/Cobimetinib. Exact *P*-values are indicated on the graphs.

© 2023 The Authors

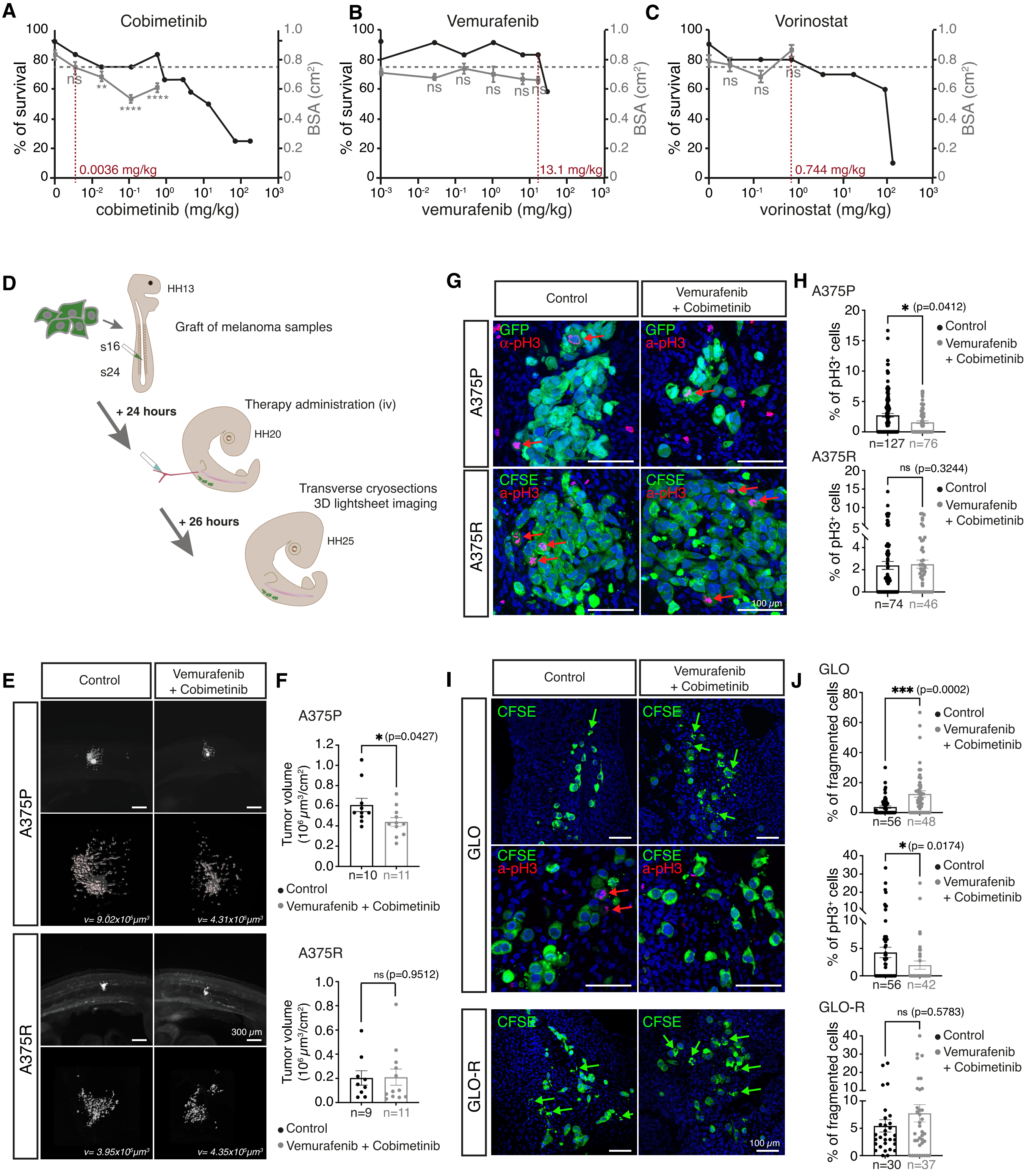

Figure EV3.

© 2023 The Authors
