# Supplementary material for: An in vivo avian model of human melanoma to perform rapid and robust preclinical studies
Source: EMBO Mol Med. 2023 Jan 24;15(3):e16629. doi: 10.15252/emmm.202216629 (PMC9994476; doi:10.15252/emmm.202216629)
Supplement: Supplementary file 7 [file EMMM-15-e16629-s004.pdf]

EMBO Molecular Medicine

An in vivo avian model of human melanoma to perform rapid and robust preclinical studies

Loraine Jarrosson, Stephane Dalle, Clélia Costechareyre, Yaqi Tang, Maxime Grimont, Maud Plaschka, Marjorie Lacourrège, Romain Teinturier, Myrtille Le Bouar, Delphine Maucort-Boulch, Anaïs Eberhardt, VALERIE CASTELLANI, Julie Caramel, and Céline Delloye-Bourgeois
DOI: 10.15252/emmm.202216629

Corresponding authors: Céline Delloye-Bourgeois (celine.delloye@univ-lyon1.fr) , Julie Caramel (Julie.caramel@lyon.unicancer.fr), VALERIE CASTELLANI (valerie.castellani@univ-lyon1.fr)

Review Timeline:

Submission Date:	22nd Jul 22
Editorial Decision:	16th Aug 22
Revision Received:	1st Dec 22
Editorial Decision:	16th Dec 22
Revision Received:	20th Dec 22
Accepted:	23rd Dec 22

Editor: Lise Roth

Transaction Report:

(Note: With the exception of the correction of typographical or spelling errors that could be a source of ambiguity, letters and reports are not edited. Depending on transfer agreements, referee reports obtained elsewhere may or may not be included in this compilation. Referee reports are anonymous unless the Referee chooses to sign their reports.)

16th Aug 2022

Dear Dr. Delloye-Bourgeois,

Thank you for the submission of your manuscript to EMBO Molecular Medicine. We have now received feedback from the three reviewers who agreed to evaluate your manuscript. As you will see from the reports below, the referees acknowledge the interest of the study and are overall supporting publication of your work pending appropriate revisions. Adequate discussion should be provided where needed, as well as clarifications regarding the imaging, statistics, and experimental protocols. However, please note that an experimental comparison of your model with PDX grown in mice (as mentioned by ref #3) will NOT be required, and this point should rather be discussed in the manuscript.

Addressing the other reviewers' concerns in full will be necessary for further considering the manuscript in our journal, and acceptance of the manuscript will entail a second round of review. EMBO Molecular Medicine encourages a single round of revision only and therefore, acceptance or rejection of the manuscript will depend on the completeness of your responses included in the next, final version of the manuscript. For this reason, and to save you from any frustrations in the end, I would strongly advise against returning an incomplete revision.

Revised manuscripts should be submitted within three months of a request for revision; they will otherwise be treated as new submissions, except under exceptional circumstances in which a short extension is obtained from the editor.

When submitting your revised manuscript, please carefully review the instructions that follow below. We perform an initial quality control of all revised manuscripts before re-review; failure to include requested items will delay the evaluation of your revision.

We require:

1) A .docx formatted version of the manuscript text (including legends for main figures, EV figures and tables). Please make sure that the changes are highlighted to be clearly visible.

2) Individual production quality figure files as .eps, .tif, .jpg (one file per figure). For guidance, download the 'Figure Guide PDF' (https://www.embopress.org/page/journal/17574684/authorguide#figureformat).

3) A .docx formatted letter INCLUDING the reviewers' reports and your detailed point-by-point responses to their comments. As part of the EMBO Press transparent editorial process, the point-by-point response is part of the Review Process File (RPF), which will be published alongside your paper.

4) A complete author checklist, which you can download from our author guidelines (https://www.embopress.org/page/journal/17574684/authorguide#submissionofrevisions). Please insert information in the checklist that is also reflected in the manuscript. The completed author checklist will also be part of the RPF.

5) It is mandatory to include a 'Data Availability' section after the Materials and Methods. Before submitting your revision, primary datasets produced in this study need to be deposited in an appropriate public database, and the accession numbers and database listed under 'Data Availability'. Please remember to provide a reviewer password if the datasets are not yet public (see https://www.embopress.org/page/journal/17574684/authorguide#dataavailability).
In case you have no data that requires deposition in a public database, please state so in this section. Note that the Data Availability Section is restricted to new primary data that are part of this study.

6) For data quantification: please specify the name of the statistical test used to generate error bars and P values, the number (n) of independent experiments (specify technical or biological replicates) underlying each data point and the test used to calculate p-values in each figure legend. The figure legends should contain a basic description of n, P and the test applied. Graphs must include a description of the bars and the error bars (s.d., s.e.m.). Please provide exact p values.

7) We would also encourage you to include the source data for figure panels that show essential data. Numerical data should be provided as individual .xls or .csv files (including a tab describing the data). For blots or microscopy, uncropped images should be submitted (using a zip archive if multiple images need to be supplied for one panel). Additional information on source data and instruction on how to label the files are available at
.

8) Our journal encourages inclusion of *data citations in the reference list* to directly cite datasets that were re-used and obtained from public databases. Data citations in the article text are distinct from normal bibliographical citations and should

directly link to the database records from which the data can be accessed. In the main text, data citations are formatted as follows: "Data ref: Smith et al, 2001" or "Data ref: NCBI Sequence Read Archive PRJNA342805, 2017". In the Reference list, data citations must be labeled with "[DATASET]". A data reference must provide the database name, accession number/identifiers and a resolvable link to the landing page from which the data can be accessed at the end of the reference. Further instructions are available at .

9) We replaced Supplementary Information with Expanded View (EV) Figures and Tables that are collapsible/expandable online. A maximum of 5 EV Figures can be typeset. EV Figures should be cited as 'Figure EV1, Figure EV2" etc... in the text and their respective legends should be included in the main text after the legends of regular figures.
- For the figures that you do NOT wish to display as Expanded View figures, they should be bundled together with their legends in a single PDF file called *Appendix*, which should start with a short Table of Content. Appendix figures should be referred to in the main text as: "Appendix Figure S1, Appendix Figure S2" etc.
- Additional Tables/Datasets should be labeled and referred to as Table EV1, Dataset EV1, etc. Legends have to be provided in a separate tab in case of .xls files. Alternatively, the legend can be supplied as a separate text file (README) and zipped together with the Table/Dataset file.
See detailed instructions here:
.

10) The paper explained: EMBO Molecular Medicine articles are accompanied by a summary of the articles to emphasize the major findings in the paper and their medical implications for the non-specialist reader. Please provide a draft summary of your article highlighting
- the medical issue you are addressing,
- the results obtained and
- their clinical impact.
This may be edited to ensure that readers understand the significance and context of the research. Please refer to any of our published articles for an example.

11) For more information: There is space at the end of each article to list relevant web links for further consultation by our readers. Could you identify some relevant ones and provide such information as well? Some examples are patient associations, relevant databases, OMIM/proteins/genes links, author's websites, etc...

12) Conflict of interest: We updated our journal's competing interests policy in January 2022 and request authors to consider both actual and perceived competing interests. Please review the policy https://www.embopress.org/competing-interests and update your competing interests if necessary.

13) Every published paper now includes a 'Synopsis' to further enhance discoverability. Synopses are displayed on the journal webpage and are freely accessible to all readers. They include a short stand first (maximum of 300 characters, including space) as well as 2-5 one-sentences bullet points that summarizes the paper. Please write the bullet points to summarize the key NEW findings. They should be designed to be complementary to the abstract - i.e. not repeat the same text. We encourage inclusion of key acronyms and quantitative information (maximum of 30 words / bullet point). Please use the passive voice. Please attach these in a separate file or send them by email, we will incorporate them accordingly.

Please also suggest a striking image or visual abstract to illustrate your article as a PNG file 550 px wide x 300-600 px high.

14) As part of the EMBO Publications transparent editorial process initiative (see our Editorial at http://embomolmed.embopress.org/content/2/9/329), EMBO Molecular Medicine will publish online a Review Process File (RPF) to accompany accepted manuscripts.
In the event of acceptance, this file will be published in conjunction with your paper and will include the anonymous referee reports, your point-by-point response and all pertinent correspondence relating to the manuscript. Let us know whether you agree with the publication of the RPF and as here, if you want to remove or not any figures from it prior to publication.
Please note that the Authors checklist will be published at the end of the RPF.

EMBO Molecular Medicine has a "scooping protection" policy, whereby similar findings that are published by others during review or revision are not a criterion for rejection. Should you decide to submit a revised version, I do ask that you get in touch after three months if you have not completed it, to update us on the status.

I look forward to receiving your revised manuscript.

Yours sincerely,

Lise Roth

Lise Roth, PhD
Senior Editor
EMBO Molecular Medicine

***** Reviewer's comments *****

Referee #1 (Comments on Novelty/Model System for Author):

Model uses embryos at developmental stages in accordance with ethical guidelines of European Directives 2010/63/EU.

Referee #1 (Remarks for Author):

In this interesting study, Jarrosson and colleagues describe a novel in vivo avian model of human melanoma that allows preclinical studies. They built upon previous models for neuroblastoma and triple negative breast cancers using the avian embryo as a recipient for graft samples from melanoma patients. The development of a new model for cancer preclinical testing, and melanoma in particular, is timely, given the high cost and long timeframe of mouse models, including patient-derived xenografts, among other issues. This model allows monitoring tumor formation and short-term treatments with clinically relevant therapies (BRAF and MEK inhibitors, HDAC inhibitors).

In general, the data is well presented, and authors test a variety of cell lines and quite a few patient-derived tumours. The main caveat, as discussed below, may be the quite short times for tumour formation and treatments used, which may preclude from observing larger, significant effects.

Specific comments.

1. Authors mention that injected cells were no longer present at the graft site. For example, Fig.1B, among others. Is it possible to show in the images both the injection site and the location at endpoint?
Similar comment for "...but not in developing sensory and sympathetic ganglia (Fig.1BC)", showing these in the image, if possible, would help.

2. Stainings should be quantified (as in Fig.2B). For example, Fig.1C-D, 1G, quantifications of Ki67, MITF+SOX10+ cells are missing.

3. Regarding statistics. In some figures it is not clear how many eggs were injected with each cell line or patient-derived material. For example, Figure 1-2 experiments. Figure 2B says "n=16 slices", from how many different embryos? In Figure 2G, each dot represents a different tumour/embryo? In Fig.2I and 2K, what statistical comparisons are being made (the asterisk or ns refers to comparing what with what)? Fig.2I and 2K may use student t-tests, but there are more than 2 groups, so a multiple comparison test (anova or similar) should be used instead. These issues should be revised throughout the manuscript.
For Fig.2I, is there any reason for the control having twice as many embryos as some of the treatments?
Also, for Fig.2A-B, text says "..fraction of A375P cells showing no or low MITF expression was significantly higher in cells disconnected from dense tumor masses..". However, the MITFneg says "ns". Perhaps rephrase?

4. Treatments were for 24h, did treatments start 48h after injection of cells? It is not clear. Did authors try longer treatments? This short treatment may be underestimating effects, for example in 2I-K, or in 3B,3 G. In Figure 3 it is not clear the length of treatments. This should be at least discussed.

5. The high variability observed in 3F-H may be also responsible for the lack of statistically significant effects in these PDXs. There are very small tumours even in the control group. Did authors try starting the treatments a bit later, so tumours are larger when treatment starts? And/or injecting more cells? Having a rapid model compatible with therapeutic decision-making would be a very strong resource, as authors discuss. However, these short tumour formation and treatment times might hinder observing larger, significant effects. This should be at least discussed.

6. In line with the above, can authors discuss whether this model may be used for longer treatments to model response to sequential therapies and, eventually, resistance? And to monitor metastasis formation?

Minor comments
- Fig.1A: Coe, NT, SO in middle diagram should be defined.

- Fig.1F: what are the dashed lines in Mel4 upper image?
- Supplementary Figure 1A-B: Labels for which cell line is which in the graph are missing.

Referee #2 (Comments on Novelty/Model System for Author):

It is not clear to me the impact of the immune system has on the claims and observations made by the authors

Referee #2 (Remarks for Author):

In this study Jarrosson et al described development of a highly efficient patient-derived xenograft model adapted to patient melanoma biopsies, using the avian embryo. In this in vivo model, they have been able to observe a fast and reproducible tumor engraftment of patient samples within the embryonic skin, preserving key molecular and phenotypic features. Through several experiments, they show that sensitivity and resistance to BRAFi/MEKi targeted therapies can be reliably modeled in these AVI-PDXTM, as well as synergies with other drugs, such as HDACi, thus proposing that it may be a valuable tool for the design of personalized medicine assays and for the evaluation of novel combination strategies

• Many research groups including (PMID: 31919461) have used zebrafish embryo as a model system to interrogate therapeutic efficacy of drugs. The authors should cite such references and discuss this in the context to their work.
• In figure 1, the authors describe the embryonic stage and territory to graft melanoma cells for successful tumor growth, but they do not describe/ provide evidence of any associated immune changes that occur with this process. Do the authors know how innate immunity is affected by this process?
• Do the authors have any data showing if the SOX10-MITF axis is maintained if A375R (resistant to PLX4032) are injected into this model system? Such resistant cell lines may also be very effective for drug efficacy studies.
• There are no ethical statements written around using avian model system. Are there no ethical considerations while using this model system?
• In addition to Ki67, it might be informative if the authors also show some other markers like p-H2AX for the tumors when using this model to test for therapeutic efficacy
• Presence of the immune system determines to a large extent the therapeutic efficacy of drugs. The authors should comment around this (immune system- their model system-presence or absence of a functional immune system and the implications of their observations that may be there if a functional immune system isn't present in their model vs real patient scenario etc)

Referee #3 (Remarks for Author):

In this study, Jarrosson et al. constructed a model system that human melanoma tissues were xenografted into chick embryo at the site of neural crest (NC). The grafted melanoma cells migrated on the dorsolateral and ventrolateral paths, subsequently reaching the developing skin or under the epidermis, like the differentiation pattern of melanoblasts. The take rates of PDX were higher than 50% except one melanoma, and the intratumoral heterogeneity (ITH) in terms of developmental states was observed especially in comparison between clustered and migrating cells. For preclinical studies, the authors first developed a procedure to identify maximum tolerated dose (MTD) in chick embryo, and then treated these PDXs with BRAF, MEK, and HDAC inhibitors. The results showed consistency with patients' short-term response to the same therapies.

Conceptually, the model system is not equivalent to disease modeling, which aims to identify driving factors of the response to the input, followed by validating the clinical relevance of the driving factors and the input (https://aacrjournals.org/cancerres/article/76/20/5921/613841/Concepts-in-Cancer-Modeling-A-Brief-HistoryCancer). Understandably, the authors intended to demonstrate that AVID-PDX is predictive of patients' responses to the small-molecule therapies. However, the modeling subject of AVID-PDX is not clear (Invasion and progression? Growth in skin environment? Acquired resistance from ITH?), so no driving factors are identified and validated, and whether the results are more predictive than the in vitro assay is not known.

Alternatively, if the authors intend to show that AVID-PDX is a system with superior tumor take rate for PDXs than immunocompromised mice, and the predictive power is no lower than the latter, the comparison between the two system is needed.

Other points:
1. What is the endpoint of the preclinical studies on this model?
2. Supp. Figure 1: the legends of GLO and GLO-R curves should be added.

Point-to-point answers to the reviewers

***** Reviewer's comments *****

Referee #1 (Comments on Novelty/Model System for Author):

Model uses embryos at developmental stages in accordance with ethical guidelines of European Directives 2010/63/EU.

Referee #1 (Remarks for Author):
In this interesting study, Jarrosson and colleagues describe a novel in vivo avian model of human melanoma that allows preclinical studies. They built upon previous models for neuroblastoma and triple negative breast cancers using the avian embryo as a recipient for graft samples from melanoma patients. The development of a new model for cancer preclinical testing, and melanoma in particular, is timely, given the high cost and long timeframe of mouse models, including patient-derived xenografts, among other issues. This model allows monitoring tumor formation and short-term treatments with clinically relevant therapies (BRAF and MEK inhibitors, HDAC inhibitors).

In general, the data is well presented, and authors test a variety of cell lines and quite a few patient-derived tumours. The main caveat, as discussed below, may be the quite short times for tumour formation and treatments used, which may preclude from observing larger, significant effects.

We thank the referee for his/her constructive comments. Please find below our answers to the issues raised in the specific and minor comments.

Specific comments.
1. Authors mention that injected cells were no longer present at the graft site. For example, Fig.1B, among others. Is it possible to show in the images both the injection site and the location at endpoint? Similar comment for "...but not in developing sensory and sympathetic ganglia (Fig.1BC)", showing these in the image, if possible, would help.
We agree with the referee that pointing at the injection site and a better annotation of the anatomic structures on the sections would definitely help. Where possible, we have added an asterisk to position the injection site and more precise legends to document the position of dorsal root ganglia (DRG) and sympathetic ganglia (SG).

2. Stainings should be quantified (as in Fig.2B). For example, Fig.1C-D, 1G, quantifications of Ki67, MITF+SOX10+ cells are missing.
We agree that several stainings were descriptive rather than used as quantitative data. We have now added a quantification of Ki67 staining (Fig. EV1A), and SOX10/MITF stainings (Fig. EV1D). Staining experiments added in the revised version of the manuscript (phospho-histone3, fragmented cells) were all quantified (Fig. EV3G-J).

3. Regarding statistics. In some figures it is not clear how many eggs were injected with each cell line or patient-derived material. For example, Figure 1-2 experiments. Figure 2B says "n=16 slices", from how many different embryos? In Figure 2G, each dot represents a different tumour/embryo? In Fig.2I and 2K, what statistical comparisons are being made (the asterisk or ns refers to comparing what with what)? Fig.2I and 2K may use student t-tests, but there are more than 2 groups, so a multiple comparison test (anova or similar) should be used instead. These issues should be revised throughout the manuscript. For Fig.2I, is there any reason for the control having twice as many embryos as some of the treatments? Also, for Fig.2A-B, text says "..fraction of A375P cells showing no or low MITF expression was significantly higher in cells disconnected from dense tumor masses..". However, the MITFneg says "ns". Perhaps rephrase?
We apologize for the lack of clarity in statistics. We have now clarified in the figures and in figure legends the comparisons made, the number of slices or embryos accordingly. More precisely:
- in Figure 2B, n=16 slices were analyzed from 3 different embryos.
- in Figure 2G: each dot represents the compaction index of one melanoma tumor -ie, one embryo- for both GLO and GLO-R tumors. Similarly, in Fig. 3F-H and Fig. EV3F, each dot represents a different tumor (and thus a different embryo).
- in Fig 2I/2K : All statistical comparisons were made for one experimental condition compared to the control (excipient). Thus, depending on data distribution and homoscedasticity of variances between two experimental

groups, we have used Student t-test or Mann-Whitney U test. We have added brackets on the graphs to highlight the experimental groups concerned by a given statistical comparison. Therefore, « ns » and asterisk always refer to statistical comparison with the control/excipient group. In this set of experiments, we have decided not to use multiple comparison tests (such as ANOVA), as each experimental group is independently analyzed and compared to the control/excipient comparison.
- in Fig 2I: the higher number of embryos in the control condition as compared to other treated conditions is due to the fact that we generated more control embryos to set up 3D imaging for GLO and GLO-R grafted embryos.
- Fig. 2AB: we thank the referee for his/her comment, the sentence was indeed not clear. We have rephrased it, in accordance with our statistical results: "Interestingly, the fraction of A375P cells showing low MITF expression was significantly higher in cells disconnected from tumor masses, suggesting a dynamic regulation of MITF expression depending on the proliferative (tumor masses) versus invasive (migrating cells) properties of melanoma cells (Fig. 2A,B)"

4. Treatments were for 24h, did treatments start 48h after injection of cells? It is not clear. Did authors try longer treatments? This short treatment may be underestimating effects, for example in 2I-K, or in 3B,3 G. In Figure 3 it is not clear the length of treatments. This should be at least discussed.
We apologize for the lack of clarity in the procedure. We have added a scheme in Fig. EV3D that better depicts the timing of the graft/treatment procedure. In all experiments presented here, treatments were administrated by IV injection 24h after engraftment. Embryos were harvested for analysis 24 hours later, thus 48 hours after the graft and after a 24 hour-treatment. Indeed, although one of our aims was to reduce the duration of in vivo studies in light of clinical needs, we have still tried longer treatments (48 hours, 96 hours) with BRAFi/MEKi. The effect of therapies on tumor volumes were highly similar to those obtained after a 24 hour-treatment. However, we completely agree that depending on the questions addressed with the graft experiments and the type(s) of analytic read-out, longer treatments could be of strong interest. We have discussed this aspect in the discussion section (lines 692-695).

5. The high variability observed in 3F-H may be also responsible for the lack of statistically significant effects in these PDXs. There are very small tumours even in the control group. Did authors try starting the treatments a bit later, so tumours are larger when treatment starts? And/or injecting more cells? Having a rapid model compatible with therapeutic decision-making would be a very strong resource, as authors discuss. However, these short tumour formation and treatment times might hinder observing larger, significant effects. This should be at least discussed.
The relative variability of tumor volumes and outlier replicates obtained in avian embryos with patient samples are inherent to the use of tumor biopsies in xenograft systems, and is in part explained by the small amount of material contained in these samples. To get around this, we engraft a maximum of embryos to obtain substantial series of tumors and generate significant experimental groups with a minimum of material. Grafting more cells would result in smaller series of tumors which would hinder the realization of statistical analyses and could even obscure interesting tendencies. Moreover, to obtain significantly bigger tumors, we would have to wait for at least one round of cell division in the tumor cell population, which would require several days and would typically be highly variable depending on the sample. Comments on the treatment times now have been added in the manuscript.

6. In line with the above, can authors discuss whether this model may be used for longer treatments to model response to sequential therapies and, eventually, resistance? And to monitor metastasis formation?
In this study, we depict the use of the avian graft model of melanoma to assess the immediate efficacy of single or combi-therapies, on primary tumors. We also show that invasive properties of melanoma cells and signs of metastatic onset are recapitulated in the grafts (Fig. 2A-G). Moreover, we show that the resistance/sensitivity profiles to BRAFi/MEKi and associated molecular markers of cell lines (A375P/A375R), patient primary cultures (GLO/GLO-R) and patient samples (Fig. 3E-H) are reproduced in avian grafts. In the scope of these revisions, we have performed additional experiments on BRAFi-sensitive A375P and BRAFi-resistant A375R pair of cell lines and could show the maintenance of both the MITF-SOX10 axis characteristics and type of response to BRAFi in graft models. Thus, we can at least model innate resistance, or in vitro acquired resistance, of melanoma cells to targeted therapies. Modeling acquired resistance in the avian graft model would require longer treatment exposure, which might be incompatible with the 21 days period before hatching, although we do not have experimental data to formally exclude this possibility. However, intravenous sequential administration of therapies in avian embryos is feasible (unpublished data) and allows mimicking therapeutic schemes used in the clinics.

We have previously conducted studies on Neuroblastoma, another type of neural crest-derived cancer, and we could indeed document the possibility to monitor metastasis onset and formation (Delloye-Bourgeois et al., 2017; Ben Amar et al., 2022), both by 3D lightsheet imaging and confocal imaging of embryonic sections, from embryonic day 5 to day 14. Thus, we believe such experiments could completely apply to melanoma, although we did not integrate this issue in the present manuscript.
We now have discussed these issues more clearly in the discussion section (lines 692-695).

Minor comments

- Fig.1A: Coe, NT, SO in middle diagram should be defined. This has been added in the figure legend.

- Fig.1F: what are the dashed lines in Mel4 upper image? Dashed lines highlight the neural tube and the notochord as anatomical landmarks on the optic transverse section. We have now mentioned it in the figure legend.

- Supplementary Figure 1A-B: Labels for which cell line is which in the graph are missing. We apologize for this omission, it has now been corrected in Fig. EV2A,B.

Referee #2 (Comments on Novelty/Model System for Author):

It is not clear to me the impact of the immune system has on the claims and observations made by the authors

Referee #2 (Remarks for Author):
In this study Jarrosson et al described development of a highly efficient patient-derived xenograft model adapted to patient melanoma biopsies, using the avian embryo. In this in vivo model, they have been able to observe a fast and reproducible tumor engraftment of patient samples within the embryonic skin, preserving key molecular and phenotypic features. Through several experiments, they show that sensitivity and resistance to BRAFi/MEKi targeted therapies can be reliably modeled in these AVI-PDXTM, as well as synergies with other drugs, such as HDACi, thus proposing that it may be a valuable tool for the design of personalized medicine assays and for the evaluation of novel combination strategies.
We thank the referee for his/her very interesting comments. Please find below our answers to the specific comments.

1. Many research groups including (PMID: 31919461) have used zebrafish embryo as a model system to interrogate therapeutic efficacy of drugs. The authors should cite such references and discuss this in the context to their work.
We agree with the referee and apologize for the lack of precise discussion on previous work made with other alternative xenograft models. Avian and zebrafish embryonic graft models share several general features among which (1) the miniaturization of xenotransplantation, (2) a high rate and fast tumor take, (3) transparency of organism at early stages allowing highly precise engraftment, and (4) possibility of short-term treatment evaluation (Xiao et al., 2020).
In particular for melanoma, several zebrafish xenograft models were designed such as transplantation of melanoma cells (cell lines, mouse PDX, but not non-expanded patient biopsies to our knowledge) in blastula, perivitelline space, yolk sac or blood stream circulation, showing efficient tumor intake and growth (Yan et al., 2019; Das et al, 2020; Haldi et al, 2006; Lee et al, 2005; Topczewska et al, 2006). Das et al also reported the relevance of the zebrafish xenograft model to evaluate and predict anti-tumor response to BRAF inhibitors. However, regarding melanoma, whether xenografting patient biopsies without any short-term pre-culture step or passage in the mouse is feasible and doable in series of animals, has not been formally documented. Differences between avian and zebrafish melanoma xenograft models also rely on technical aspects, that in our opinion could have important impacts on functional analyses, such as incubation of zebrafish larvae at low temperature (34°C) inconsistent with human physiology, or water delivery of drug candidates. Solubility and absorptive ability of drugs added in water varies. However, such an administration mode makes the zebrafish embryo particularly suited for high throughput genetic and chemical screening in the drug discovery field.

We have now better discussed the complementary features of these xenograft melanoma models in the discussion section (lines 665-684).

Xiao et al, 2020. Zebrafish xenografts for drug discovery and personalized medicine. Trends in cancer (Cell).
Yan et al, 2019. Visualizing Engrafted Human Cancer and Therapy Responses in Immunodeficient Zebrafish. Cell 2019 vol: 177 (7) pp: 1903-1914.e14
Das et al, 2020 AXL and CAV-1 play a role for MTH1 inhibitor TH1579 sensitivity in cutaneous malignant melanoma. Cell death and differentiation (2020).
Haldi et al, 2006. Human melanoma cells transplanted into zebrafish proliferate, migrate, produce melanin, form masses and stimulate angiogenesis in zebrafish. Angiogenesis
Lee et al; 2005. The fate of human malignant melanoma cells transplanted into zebrafish embryos: Assessment of migration and cell division in the absence of tumor formation. Dev. Dyn. 2005
Topczewska et al, 2006. Embryonic and tumorigenic pathways converge via Nodal signaling: Role in melanoma aggressiveness. Nat. Med. 2006

2. In figure 1, the authors describe the embryonic stage and territory to graft melanoma cells for successful tumor growth, but they do not describe/ provide evidence of any associated immune changes that occur with this process. Do the authors know how innate immunity is affected by this process?
The full graft/treatment/analytic procedure depicted here happens between embryonic day 2 (E2, HH14) and embryonic day 4 (E4, HH25). At this early stage of development, chicken embryos show neither innate nor adaptative immunity, which may provide an immunopermissive microenvironment for xenotransplantation. But Several studies and reviews described the spatiotemporal ontogeny of chicken immune cells during embryogenesis. Dòra et al have shown that only few immature and non-functional $CD45^+$ cells spread within the chicken embryo at early developmental stages (Dòra et al., 2019). In regards to adaptative immunity, B and T cells start colonizing primary and secondary lymphoid organs between E10 and E14, where they become fully immune-competent several days post-hatching (Alkie et al, 2019; Garcia et al., 2021).
Although we cannot rule out that innate immunity and early immature $CD45^+$ cells are affected/undergo modifications upon the graft procedure (between E2 and E4), we hypothesize that such an immunocompromise tumor environment in early chicken embryo developmental stages likely participates to high tumor intake rate observed in our models. This is now discussed in lines 636-640.

Dòra et al, 2017. Ontogeny of ramified CD45 cells in chick embryo and their contribution to bursal secretory dendritic cells. Cell tissue Res
Alkie et al, 2019. Development of innate immunity in chicken embryos and newly hatched chicks: a disease control perspective. Avian pathology.
Garcia P, Wang Y, Viallet J and Macek Jilkova Z (2021) The Chicken Embryo Model: A Novel and Relevant Model for Immune-Based Studies. Front. Immunol. 12:791081. doi: 10.3389/fimmu.2021.791081

3. Do the authors have any data showing if the SOX10-MITF axis is maintained if A375R (resistant to PLX4032) are injected into this model system? Such resistant cell lines may also be very effective for drug efficacy studies.
We have performed some additional experiments with the A375R cells to answer this point. The PLX4032-resistant A375R cell line was engrafted in series of chicken embryos. We analyzed the expression level of both SOX10 and MITF proteins by immunofluorescence in grafted A375R cells as compared to A375P cells (Fig. EV1C,D). In accordance with *in vitro* expression of SOX10 (Western Blot, Fig. EV1B) and MITF (Richard et al., 2016) in A375P and A375T cells, we observed that MITF expression drastically dropped in A375R-xenografts in comparison with A375P-xenografts, in which 50% of those cells expressed MITF. Decrease in MITF associated with resistance to BRAF inhibitors is a commonly observed feature in BRAF-mutated melanoma (Müller et al, 2015). As suggested by the referee, we compared the efficacy of BRAFi/MEKi on A375P and A375R avian xenografts, and could confirm that, as opposed to A375P, A375R tumor volumes were not impacted by the treatment (Fig. EV3E,F). Together with data obtained with GLO-R cells and with patient samples showing distinct degrees of response to BRAFi/MEKi in clinics, these data strengthen the idea that melanoma cells retain their resistance/sensitivity profile to BRAFi/MEKi once grafted in chicken embryos.

Müller et al, 2015. Low MITF/AXL ratio predicts early resistance to multiple targeted drugs in melanoma. *Nature communications*

4. There are no ethical statements written around using avian model system. Are there no ethical considerations while using this model system?

The use of the avian embryo as a model system was examined by the revised ethical guidelines of the European Directive EU 2013 86/609/EEC. The latter states that using the avian embryo during the first 2 weeks of its development (from E1 to E14) conforms with the 3R principles, and doesn't require any ethical statement. In our case, all experiments are performed before E14 explaining the absence of ethical statement and also ensuring high flexibility in the use of this model system.

5. In addition to Ki67, it might be informative if the authors also show some other markers like p-H2AX for the tumors when using this model to test for therapeutic efficacy

We have now provided additional data to better relate variations of tumor volumes with molecular markers when assessing therapeutic efficacy.

As suggested, we performed p-H2AX stainings on sections of GLO/GLO-R and A375P/A375R xenografts, treated with excipient or a combination of BRAFi/MEKi (not shown). However, very few p-H2AX positive cells were detected in both conditions, whatever the cell lines, precluding the realization of relevant statistical tests. We previously showed in our avian model of breast cancer that treatment with gemcitabine/carboplatin triggered an increase in the number of p-H2AX positive cells, suggesting that early steps of DNA damage response occurred in TNBC xenografts after treatment (Jarrosson et al., 2021). This might be due to different modes of action of BRAFi/MEKi and chemotherapeutic targeting agents. According to their mode of action, BRAF and MEK inhibitors are unlikely to induce DNA damage response.

To overcome this issue, we analyzed the expression of another marker, phospho-histone-3 (PH3), that labels mitotic cells (Fig EV3G-J) in sections of GLO/GLO-R and A375P/A375R xenografts, treated with excipient or a combination of BRAFi/MEKi. We observed that the number of $PH3^+$ cells in both GLO and A375P xenografts treated with BRAFi/MEKi was significantly decreased as compared to the excipient condition, in accordance with the effect on tumor volumes. In contrast, A375R xenografts did not show any variation in the fraction of PH3+ cells with BRAFi/MEKi treatment. Unfortunately, we could not perform this analysis on GLO-R xenografts as the fraction of PH3+ cells was too small to perform relevant statistical comparisons. Hence, we completed the GLO/GLO-R xenografts comparison by quantifying the fraction of cells showing typical signs of cell death -ie, cell fragmentation, highlighted by $CFSE^+$ multiple cytoplasmic fragments surrounding the nucleus-. Using this criteria, we could observe an increase in the fraction of fragmented cells in GLO xenografts treated with BRAFi/MEKi, but not in GLO-R xenografts in accordance with the absence of BRAFi/MEKi treatment on GLO-R tumor volumes. We hope that these additional quantifications will better document the effect of BRAFi/MEKi on melanoma cells grafted in the avian embryo.

6. Presence of the immune system determines to a large extent the therapeutic efficacy of drugs. The authors should comment around this (immune system- their model system-presence or absence of a functional immune system and the implications of their observations that may be there if a functional immune system isn't present in their model vs real patient scenario etc)

We completely agree with this comment and already commented this issue in point #2. We provided additional discussion regarding the immune status of avian embryos in the revised manuscript (lines 636-640). In particular, from E2 to E4, the avian immune system is fully immature. Thus, when grafting melanoma cell lines in avian embryos, the observations / analyses extracted from our model system do not integrate the potential involvement of the immune system. However, the scenario may be different when using human tumor samples. Indeed, those samples may contain infiltrated immune cells, that following our dissociation and graft procedure -aiming at preserving intra-tumor heterogeneity- are co-implanted in avian embryos together with tumor cells. However, we have not performed an exhaustive study on the fraction of immune cells that could be co-implanted with tumor cells from patient samples in the present study. Further work dedicated to this issue will definitively help characterizing the immune landscape of patient-derived xenografts in the avian embryonic model and may lay the bases for implementing our paradigm to better fit with immune-related challenges.

Referee #3 (Remarks for Author):

In this study, Jarrosson et al. constructed a model system that human melanoma tissues were xenografted into

chick embryo at the site of neural crest (NC). The grafted melanoma cells migrated on the dorsolateral and ventrolateral paths, subsequently reaching the developing skin or under the epidermis, like the differentiation pattern of melanoblasts. The take rates of PDX were higher than 50% except one melanoma, and the intratumoral heterogeneity (ITH) in terms of developmental states was observed especially in comparison between clustered and migrating cells. For preclinical studies, the authors first developed a procedure to identify maximum tolerated dose (MTD) in chick embryo, and then treated these PDXs with BRAF, MEK, and HDAC inhibitors. The results showed consistency with patients' short-term response to the same therapies.

Conceptually, the model system is not equivalent to disease modeling, which aims to identify driving factors of the response to the input, followed by validating the clinical relevance of the driving factors and the input (https://aacrjournals.org/cancerres/article/76/20/5921/613841/Concepts-in-Cancer-Modeling-A-Brief-HistoryCancer). Understandably, the authors intended to demonstrate that AVI-PDX is predictive of patients' responses to the small-molecule therapies. However, the modeling subject of AVI-PDX is not clear (Invasion and progression? Growth in skin environment? Acquired resistance from ITH?), so no driving factors are identified and validated, and whether the results are more predictive than the in vitro assay is not known.
Alternatively, if the authors intend to show that AVI-PDX is a system with superior tumor take rate for PDXs than immunocompromised mice, and the predictive power is no lower than the latter, the comparison between the two system is needed.

We thank the reviewer for his/her comment and apologize if the modeling subject(s) of our approach was not exposed in a clear manner. Our purpose was to propose an alternative to melanoma patient-derived xenograft systems that would be ethical, fast and efficient without affecting molecular features that are known to be key in melanoma cell behaviors (proliferation, invasion) and response to targeted therapies. Potential melanoma driving factors were neither functionally examined nor challenged here.
In line with this comment and advise from the editorial board, we have added comments in the discussion section on key specificities of the AVI-PDX as compared to mouse PDX model systems in particular in terms of tumor take rate (lines 665-684). We hope that this modification will make our message, and the particularities of the AVI-PDX^{TM} clearer. In particular we mention differences in:

- Tumor take rate: 60% in mouse versus 100% in the AVI-PDX
- Amount of material needed: 2 to 3 grafts per biopsy in mouse versus 10 to 40 grafts per biopsy in the AVI-PDX
- Time to obtain tumor take: mean of 10 weeks in mouse versus 24 hours in the AVI-PDX
- Imaging techniques: imaging at single cell resolution possible only in the AVI-PDX
- Ethics: use of the avian embryo at early developmental stages (from E1 to E14) fully meets the 3R principles, according to the revised EU directive 2013 86/609/EEC.

Other points

1. What is the endpoint of the preclinical studies on this model?
The use of the avian embryo as a model system was examined by the revised ethical guidelines of the European Directive EU 2013 86/609/EEC. The latter states that using the avian embryo during the 2 first weeks of its development (from E1 to E14) conforms with the 3R principles, and doesn't require any ethical statement. In our case, all experiments are performed before E14 explaining the absence of ethical statement and reference to a defined endpoint. For preclinical studies that would require using the avian model after E14, an ethical statement and defined endpoint would be mandatory.

2. Supp. Figure 1: the legends of GLO and GLO-R curves should be added.
We apologize for this omission, it has now been corrected in Fig. EV2A,B.

16th Dec 2022

Dear Dr. Delloye-Bourgeois,

Thank you for the submission of your revised manuscript to EMBO Molecular Medicine. We have now received the enclosed reports from the three initial referees. As you will see below, while referees #1 and #2 are satisfied with the revision, referee #3 still raises a few concerns. We further discussed your manuscript and the referee's comments with my colleagues, and agreed that these concerns could be addressed in writing only (by adding discussion in the text).
Moreover, please address the following editorial points:

1/ Main manuscript text:
- Please accept all changes and only keep in track changes mode any new modification.
- Please provide up to 5 keywords.
- Material and methods:
o Cell culture: Please indicate whether the cells were tested for mycoplasma contamination.
o Human samples: Please that the experiments conformed to the principles set out in the WMA Declaration of Helsinki and the Department of Health and Human Services Belmont Report.
- Data Availability Section: Thank you for depositing your data in a public repository. Please note that the data must be public before acceptance of the manuscript.
- Please note that all corresponding authors are required to supply an ORCID ID for their name upon submission of a revised manuscript (currently missing for V. Castellani). We note that you currently have together with you, a total of 3 co-corresponding authors. Is that correct? Do you confirm equal contribution of these 3 people, able to take full responsibility for the paper and its content?

2/ Figures:
- Table EV1: please remove the legend from the manuscript file and add it to the table file.

3/ Thank you for providing Source Data. Please upload them as one file per figure.

4/ Thank you for providing a synopsis. The short stand first should be maximum 300 characters, including space. The bullet points should not repeat the stand first. Please adjust accordingly.

5/ As part of the EMBO Publications transparent editorial process initiative (see our Editorial at http://embomolmed.embopress.org/content/2/9/329), EMBO Molecular Medicine will publish online a Review Process File (RPF) to accompany accepted manuscripts.
This file will be published in conjunction with your paper and will include the anonymous referee reports, your point-by-point response and all pertinent correspondence relating to the manuscript. Let us know whether you agree with the publication of the RPF and as here, if you want to remove or not any figures from it prior to publication.
Please note that the Authors checklist will be published at the end of the RPF.

I look forward to receiving your revised manuscript.

With kind regards,

Lise

Lise Roth, PhD
Senior Editor
EMBO Molecular Medicine

***** Reviewer's comments *****

Referee #1 (Comments on Novelty/Model System for Author):

Model uses embryos at developmental stages in accordance with ethical guidelines of European Directives 2010/63/EU. Tumours are shown to develop faster than in mice

Referee #1 (Remarks for Author):

The authors have addressed all my comments, I support publication.

Referee #2 (Remarks for Author):

I thank the authors were addressing all my review comments to my satisfaction.

Referee #3 (Comments on Novelty/Model System for Author):

Please see my comments to the authors.

Referee #3 (Remarks for Author):

For using in preclinical studies or drug screening, the tumor model needs to be traceable and reproducible. In the AVI-PDX model, the tumors are measured at endpoint only (i.e. after study stopped) by histological analysis. Moreover, whether the tumor tissue could be expanded for cryopreservation and repeating the study is not mentioned. When these two points are addressed, a comparison with organoid culture system (e.g. https://www.sciencedirect.com/science/article/pii/S2589004220305988) should be discussed.

Point-to-point answer to the referees – R2

***** Reviewer's comments *****

Referee #1 (Comments on Novelty/Model System for Author):

Model uses embryos at developmental stages in accordance with ethical guidelines of European Directives 2010/63/EU.
Tumours are shown to develop faster than in mice

Referee #1 (Remarks for Author):

The authors have addressed all my comments, I support publication.

Referee #2 (Remarks for Author):

I thank the authors were addressing all my review comments to my satisfaction.

We thank Referee#1 and Referee#2 for their comments and support for publication of our manuscript.

Referee #3 (Remarks for Author):

For using in preclinical studies or drug screening, the tumor model needs to be traceable and reproducible. In the AVI-PDX model, the tumors are measured at endpoint only (i.e. after study stopped) by histological analysis. Moreover, whether the tumor tissue could be expanded for cryopreservation and repeating the study is not mentioned. When these two points are addressed, a comparison with organoid culture system (e.g. https://www.sciencedirect.com/science/article/pii/S2589004220305988) should be discussed.

We thank the referee for his/her advices and have added to the discussion two paragraphs related to these concerns. We hope that these additional comments will meet the referee's expectations and better document the comparison and possible synergies in between patient-derived models.

Lines 287-291: "Lightsheet 3D imaging of the tumors implies that quantifications are made at endpoint only, precluding time frame analyses of tumor growth, which can rather be evaluated by measuring tumor volumes in independent, statistically relevant, experimental groups stopped at different time points."

Lines 300-312: "We have previously documented the possibility to harvest and sort viable human tumor cells from avian tissues days after the graft (Delloye-Bourgeois et al, 2017; Jarrosson et al, 2021). However, whether such post-graft material can be expanded and/or cryopreserved to perform serial engraftment in the avian embryo, although theoretically feasible, needs further experimental evaluation. The latter potential limitation is indeed circumvented with patient-derived organoids (PDO), that hold the advantage to be expandable and cryopreservable, allowing traceability and repeatability of preclinical experiments. While melanoma PDO may be too long to establish for personalized medicine purposes (Ronteix et al, 2022), using them as high-quality starting material to perform xenografts in animal models has proven efficient (Vilgelm et al, 2020) and would be a valuable approach to combine with the AVI-PDX™ in vivo paradigm and to optimize its use for preclinical studies."

23rd Dec 2022

Dear Dr. Delloye-Bourgeois, dear Celine,

Thank you for submitting your revised files. I am pleased to inform you that your manuscript is accepted for publication and is now being sent to our publisher to be included in the next available issue of EMBO Molecular Medicine.

Please read below for additional IMPORTANT information regarding your article, its publication and the production process.

Congratulations on your interesting work!

With kind regards, and my best wishes for the holiday season,

Lise

Lise Roth, Ph.D
Senior Editor
EMBO Molecular Medicine

Follow us on Twitter @EmboMolMed
Sign up for eTOCs at embopress.org/alertsfeeds

EMBO Press Author Checklist

Corresponding Author Name: Caramel & Delloye-Bourgeois
Journal Submitted to: Embo Molecular Medicine
Manuscript Number: EMM-2022-16629

USEFUL LINKS FOR COMPLETING THIS FORM
The EMBO Journal - Author Guidelines
EMBO Reports - Author Guidelines
Molecular Systems Biology - Author Guidelines
EMBO Molecular Medicine - Author Guidelines

Reporting Checklist for Life Science Articles (updated January 2022)
This checklist is adapted from Materials Design Analysis Reporting (MDAR) Checklist for Authors. MDAR establishes a minimum set of requirements in transparent reporting in the life sciences (see Statement of Task: 10.31222/osf.io/9sm4x). Please follow the journal's guidelines in preparing your manuscript.
Please note that a copy of this checklist will be published alongside your article.

Abridged guidelines for figures
1. Data
The data shown in figures should satisfy the following conditions:
- the data were obtained and processed according to the field's best practice and are presented to reflect the results of the experiments in an accurate and unbiased manner.
- ideally, figure panels should include only measurements that are directly comparable to each other and obtained with the same assay.
- plots include clearly labeled error bars for independent experiments and sample sizes. Unless justified, error bars should not be shown for technical replicates.
- if n<5, the individual data points from each experiment should be plotted. Any statistical test employed should be justified.
- Source Data should be included to report the data underlying figures according to the guidelines set out in the authorship guidelines on Data Presentation.

2. Captions
Each figure caption should contain the following information, for each panel where they are relevant:
- a specification of the experimental system investigated (eg cell line, species name).
- the assay(s) and method(s) used to carry out the reported observations and measurements.
- an explicit mention of the biological and chemical entity(ies) that are being measured.
- an explicit mention of the biological and chemical entity(ies) that are altered/varied/perturbed in a controlled manner.
- the exact sample size (n) for each experimental group/condition, given as a number, not a range;
- a description of the sample collection allowing the reader to understand whether the samples represent technical or biological replicates (including how many animals, litters, cultures, etc.).
- a statement of how many times the experiment shown was independently replicated in the laboratory.
- definitions of statistical methods and measures:
  - common tests, such as t-test (please specify whether paired vs. unpaired), simple χ2 tests, Wilcoxon and Mann-Whitney tests, can be unambiguously identified by name only, but more complex techniques should be described in the methods section;
  - are tests one-sided or two-sided?
  - are there adjustments for multiple comparisons?
  - exact statistical test results, e.g., P values = x but not P values < x;
  - definition of 'center values' as median or average;
  - definition of error bars as s.d. or s.e.m.

Please complete ALL of the questions below.
Select "Not Applicable" only when the requested information is not relevant for your study.

Materials

Newly Created Materials	Information included in the manuscript?	In which section is the information available? (Reagents and Tools Table, Materials and Methods, Figures, Data Availability Section)
New materials and reagents need to be available; do any restrictions apply?	Not Applicable	

Antibodies	Information included in the manuscript?	In which section is the information available? (Reagents and Tools Table, Materials and Methods, Figures, Data Availability Section)
For **antibodies** provide the following information:<br>- Commercial antibodies: RRID (if possible) or supplier name, catalogue number and or/clone number<br>- Non-commercial: RRID or citation	Yes	Material and Methods

DNA and RNA sequences	Information included in the manuscript?	In which section is the information available? (Reagents and Tools Table, Materials and Methods, Figures, Data Availability Section)
**Short novel DNA or RNA including primers, probes**: provide the sequences.	Not Applicable	

Cell materials	Information included in the manuscript?	In which section is the information available? (Reagents and Tools Table, Materials and Methods, Figures, Data Availability Section)
**Cell lines:** Provide species information, strain. Provide accession number in repository **OR** supplier name, catalog number, clone number, and/**OR** RRID.	Yes	Material and Methods
**Primary cultures:** Provide species, strain, sex of origin, genetic modification status.	Yes	Material and Methods
Report if the cell lines were recently **authenticated** (e.g., by STR profiling) and tested for mycoplasma contamination.	Yes	Material and Methods

Experimental animals	Information included in the manuscript?	In which section is the information available? (Reagents and Tools Table, Materials and Methods, Figures, Data Availability Section)
**Laboratory animals or Model organisms:** Provide species, strain, sex, age, genetic modification status. Provide accession number in repository **OR** supplier name, catalog number, clone number, **OR** RRID.	Yes	Material and Methods
**Animal observed in or captured from the field:** Provide species, sex, and age where possible.	Not Applicable	
Please detail **housing and husbandry conditions**.	Yes	Material and Methods

Plants and microbes	Information included in the manuscript?	In which section is the information available? (Reagents and Tools Table, Materials and Methods, Figures, Data Availability Section)
**Plants:** provide species and strain, ecotype and cultivar where relevant, unique accession number if available, and source (including location for collected wild specimens).	Not Applicable	
**Microbes:** provide species and strain, unique accession number if available, and source.	Not Applicable	

Human research participants	Information included in the manuscript?	In which section is the information available? (Reagents and Tools Table, Materials and Methods, Figures, Data Availability Section)
If collected and within the bounds of privacy constraints report on age, sex and gender or ethnicity for all study participants.	Yes	Material and Methods and Table EV1

Core facilities	Information included in the manuscript?	In which section is the information available? (Reagents and Tools Table, Materials and Methods, Figures, Data Availability Section)
If your work benefited from core facilities, was their service mentioned in the acknowledgments section?	Yes	Acknowledgements

Design

Study protocol	Information included in the manuscript?	In which section is the information available? (Reagents and Tools Table, Materials and Methods, Figures, Data Availability Section)
If study protocol has been **pre-registered, provide DOI in the manuscript**. For clinical trials, provide the trial registration number **OR** cite DOI.	Not Applicable	
Report the **clinical trial registration number** (at ClinicalTrials.gov or equivalent), where applicable.	Yes	Material and Methods

Laboratory protocol	Information included in the manuscript?	In which section is the information available? (Reagents and Tools Table, Materials and Methods, Figures, Data Availability Section)
Provide DOI OR other citation details if **external detailed step-by-step protocols** are available.	Not Applicable	

Experimental study design and statistics	Information included in the manuscript?	In which section is the information available? (Reagents and Tools Table, Materials and Methods, Figures, Data Availability Section)
Include a statement about **sample size** estimate even if no statistical methods were used.	Yes	Material and Methods
Were any steps taken to minimize the effects of subjective bias when allocating animals/samples to treatment (e.g. **randomization procedure**)? If yes, have they been described?	Yes	Material and Methods
Include a statement about **blinding** even if no blinding was done.	Yes	Material and Methods
Describe **inclusion/exclusion criteria** if samples or animals were excluded from the analysis. Were the criteria pre-established?<br><br>If sample or data points were omitted from analysis, report if this was due to attrition or intentional exclusion and provide justification.	Yes	Material and Methods
For every figure, are **statistical tests** justified as appropriate? Do the data meet the assumptions of the tests (e.g., normal distribution)? Describe any methods used to assess it. Is there an estimate of variation within each group of data? Is the variance similar between the groups that are being statistically compared?	Yes	Material and Methods and Figure legends

Sample definition and in-laboratory replication	Information included in the manuscript?	In which section is the information available? (Reagents and Tools Table, Materials and Methods, Figures, Data Availability Section)
In the figure legends: state number of times the experiment was **replicated** in laboratory.	Yes	Material and Methods
In the figure legends: define whether data describe **technical or biological replicates**.	Yes	Figure legends

Ethics

Ethics	Information included in the manuscript?	In which section is the information available? (Reagents and Tools Table, Materials and Methods, Figures, Data Availability Section)
Studies involving **human participants**: State details of **authority granting ethics approval** (IRB or equivalent committee(s), provide reference number for approval.	Yes	Material and Methods
Studies involving **human participants**: Include a statement confirming that **informed consent** was obtained from all subjects and that the experiments conformed to the principles set out in the WMA Declaration of Helsinki and the Department of Health and Human Services Belmont Report.	Yes	Material and Methods
Studies involving **human participants:** For publication of **patient photos**, include a statement confirming that consent to publish was obtained.	Not Applicable	
Studies involving experimental **animals**: State details of **authority granting ethics approval** (IRB or equivalent committee(s), provide reference number for approval. Include a statement of compliance with ethical regulations.	Not Applicable	
Studies involving **specimen and field samples:** State if relevant **permits** obtained, provide details of authority approving study; if none were required, explain why.	Not Applicable	

Dual Use Research of Concern (DURC)	Information included in the manuscript?	In which section is the information available? (Reagents and Tools Table, Materials and Methods, Figures, Data Availability Section)
Could your study fall under dual use research restrictions? Please check biosecurity documents and list of **select agents and toxins** (CDC): https://www.selectagents.gov/sat/list.htm	Not Applicable	
If you used a select agent, is the security level of the lab appropriate and reported in the manuscript?	Not Applicable	
If a study is subject to dual use research of concern regulations, is the name of the **authority granting approval and reference number** for the regulatory approval provided in the manuscript?	Not Applicable	

Reporting

The MDAR framework recommends adoption of discipline-specific guidelines, established and endorsed through community initiatives. Journals have their own policy about requiring specific guidelines and recommendations to complement MDAR.

Adherence to community standards	Information included in the manuscript?	In which section is the information available? (Reagents and Tools Table, Materials and Methods, Figures, Data Availability Section)
State if relevant guidelines or checklists (e.g., **ICMJE, MIBBI, ARRIVE, PRISMA**) have been followed or provided.	Not Applicable	
For **tumor marker prognostic studies,** we recommend that you follow the **REMARK** reporting guidelines (see link list at top right). See author guidelines, under 'Reporting Guidelines'. Please confirm you have followed these guidelines.	Not Applicable	
For **phase II and III randomized controlled trials**, please refer to the **CONSORT** flow diagram (see link list at top right) and submit the CONSORT checklist (see link list at top right) with your submission. See author guidelines, under 'Reporting Guidelines'. Please confirm you have submitted this list.	Not Applicable	

Data Availability

Data availability	Information included in the manuscript?	In which section is the information available? (Reagents and Tools Table, Materials and Methods, Figures, Data Availability Section)
Have **primary datasets** been deposited according to the journal's guidelines (see 'Data Deposition' section) and the respective accession numbers provided in the Data Availability Section?	Yes	Data Availability
Were **human clinical and genomic datasets** deposited in a public access-controlled repository in accordance to ethical obligations to the patients and to the applicable consent agreement?	Not Applicable	
Are **computational models** that are central and integral to a study available without restrictions in a machine-readable form? Were the relevant accession numbers or links provided?	Not Applicable	
If publicly available data were reused, provide the respective **data citations in the reference list**.	Not Applicable	
